# HIV-1 Protease and Reverse Transcriptase Inhibitory Activities of *Curcuma aeruginosa* Roxb. Rhizome Extracts and the Phytochemical Profile Analysis: In Vitro and In Silico Screening

**DOI:** 10.3390/ph14111115

**Published:** 2021-10-31

**Authors:** Chanin Sillapachaiyaporn, Panthakarn Rangsinth, Sunita Nilkhet, Nuntanat Moungkote, Siriporn Chuchawankul

**Affiliations:** 1 Program in Clinical Biochemistry and Molecular Medicine, Department of Clinical Chemistry, Faculty of Allied Health Sciences, Chulalongkorn University, Bangkok 10330, Thailand; 6271002637@student.chula.ac.th (C.S.); 6273006537@student.chula.ac.th (S.N.); 2 Department of Transfusion Medicine and Clinical Microbiology, Faculty of Allied Health Sciences, Chulalongkorn University, Bangkok 10330, Thailand; panthakarn.r@chula.ac.th (P.R.); Nuntanat.m@chula.ac.th (N.M.); 3 Immunomodulation of Natural Products Research Group, Faculty of Allied Health Sciences, Chulalongkorn University, Bangkok 10330, Thailand

**Keywords:** *Curcuma aeruginosa* Roxb., HIV-1 protease inhibitor, HIV-1 reverse transcriptase inhibitor, immunodeficiency virus, AIDS, virtual screening, ADMET analysis, drug discovery

## Abstract

Human immunodeficiency virus type-1 (HIV-1) infection causes acquired immunodeficiency syndrome (AIDS). Currently, several anti-retroviral drugs are available, but adverse effects of these drugs have been reported. Herein, we focused on the anti-HIV-1 activity of *Curcuma aeruginosa* Roxb. (CA) extracted by hexane (CA-H), ethyl acetate (CA-EA), and methanol (CA-M). The in vitro HIV-1 protease (PR) and HIV-1 reverse transcriptase (RT) inhibitory activities of CA extracts were screened. CA-M potentially inhibited HIV-1 PR (82.44%) comparable to Pepstatin A (81.48%), followed by CA-EA (67.05%) and CA-H (47.6%), respectively. All extracts exhibited moderate inhibition of HIV-1 RT (64.97 to 76.93%). Besides, phytochemical constituents of CA extracts were identified by GC-MS and UPLC-HRMS. Fatty acids, amino acids, and terpenoids were the major compounds found in the extracts. Furthermore, drug-likeness parameters and the ability of CA-identified compounds on blocking of the HIV-1 PR and RT active sites were in silico investigated. Dihydroergocornine, 3β,6α,7α-trihydroxy-5β-cholan-24-oic acid, and 6β,11β,16α,17α,21-Pentahydroxypregna-1,4-diene-3,20-dione-16,17-acetonide showed strong binding affinities at the active residues of both HIV-1 PR and RT. Moreover, antioxidant activity of CA extracts was determined. CA-EA exhibited the highest antioxidant activity, which positively related to the amount of total phenolic content. This study provided beneficial data for anti-HIV-1 drug discovery from CA extracts.

## 1. Introduction

Acquired immunodeficiency syndrome (AIDS) is caused by the infection of the human immunodeficiency virus (HIV). Both HIV-1 and HIV-2 are found in HIV-infected patients, but HIV-1 infection shows more severe clinical pathology and progression of disease than HIV-2 infection [1]. HIV-1 can infect the immune cells and, use the benefit of the host cells to replicate itself, and destroy the host cells. These processes cause immunocompromised hosts, leading to the infection of opportunistic pathogens such as *Mycobacterium tuberculosis* and finally death [2]. In the HIV-1 life cycle, the virus uses three important enzymes for its replication, including integrase, reverse transcriptase (HIV-1 RT), and protease (HIV-1 PR) [3,4,5]. Thus, the blocking of these enzymes can inhibit viral replication. The enzymatic inhibition is an important strategic approach for anti-HIV drug discovery. Now, many HIV-1 inhibitors are available, but the adverse effects of these drugs are still reported [6,7,8]. Therefore, a discovery of novel compounds from plant-based sources is an important research to help HIV-1 infected patients.

A combination of three antiretroviral therapy, called highly active antiretroviral therapy (HAART), is composed of two nucleoside RT inhibitors plus either a non-nucleoside RT or a PR inhibitor [9]. It has been reported that using HAART dramatically decreased mortality rate of HIV infected patients [10]. HIV-1 RT has two enzymatic activities: ribonuclease H (RNase H) and DNA polymerase activities. RNase H plays role in RNA template degradation, while DNA polymerase synthesizes DNA strand. These two enzymatic activities of HIV-1 RT cooperate in the reverse transcription step of HIV-1 replication [11]. For HIV-1 PR, it plays an important role in the viral maturation step by cleaving polyproteins to functional proteins [4]. The successful drug targets that are generally used for HIV treatment are PR and RT [3]. Therefore, in this study, we focused on HIV-1 PR and RT inhibitions.

At present, many studies have focused on anti-HIV-1 activity from natural products [12,13,14,15]. *Curcuma aeruginosa* Roxb. (CA) is known as “Wan ma ha mek” in Thai. It is a Zingiberaceae plant that has been used as traditional medicine. Pharmaceutical studies demonstrated that CA exhibited vast medicinal properties such as immunomodulatory [16], anti-androgenic [17,18], anti-nociceptive [19], anti-microbial [20], and anti-viral activities [21]. Interestingly, a previous study reported that water extract of CA rhizomes exhibits inhibition against HIV-1 replication in MT-4 cells [21]. However, the effects of CA phytochemical compounds on HIV-1 PR and HIV-1 RT inhibitory activities and their mechanisms of action have never been investigated.

Herein, we aimed to determine in vitro inhibitory activity of CA extracts: hexane (CA-H), ethyl acetate (CA-EA), and methanol (CA-M) extracts on HIV-1 PR and HIV-1 RT. In addition, phytochemical constituents in the extracts were identified using GC-MS and UPLC-HRMS. Besides, in silico screening of bioactive compounds on HIV-1 PR and HIV-1 RT was studied by molecular docking via AutoDock 4.2 and AutoDock Vina programs. Moreover, total phenolic content and antioxidant activity of CA extracts were measured. The findings from this study provide beneficial information for anti-HIV-1 drug discovery from CA extracts.

## 2. Results

### 2.1. Plant Extract Preparation

**Figure 1:**
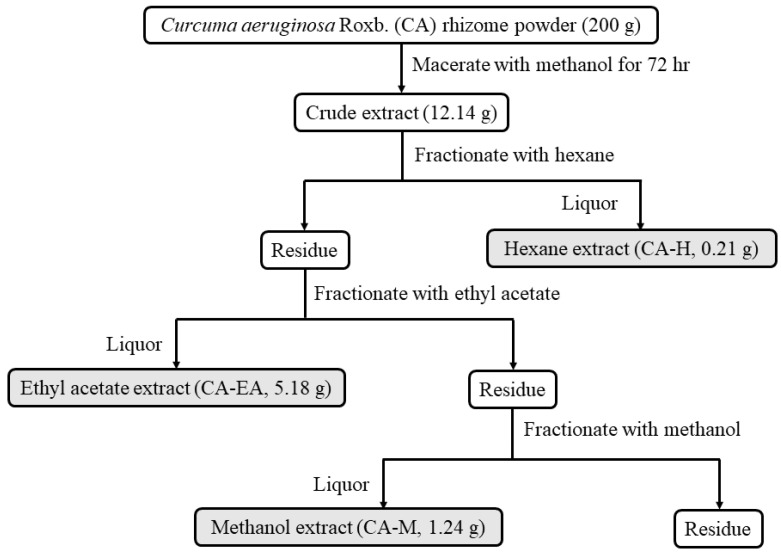
Schematic of *Curcuma aeruginosa* Roxb. (CA) extraction and fractionation. Rhizomes of *Curcuma aeruginosa* Roxb. were extracted with methanol then sequentially fractionated with hexane (CA-H), ethyl acetate (CA-EA), and methanol (CA-M), respectively.

*Curcuma aeruginosa* Roxb. (CA) extracts were prepared by the procedure as shown in Figure 1. Three crude extracts were provided including hexane (CA-H), ethyl acetate (CA-EA), and methanol (CA-M) extracts. The extraction yields of KP-H, KP-EA, and KP-M were 0.11, 2.59, and 0.62% (*w/w*), respectively.

### 2.2. HIV-1 Protease Inhibitor Screening

**Figure 2:**
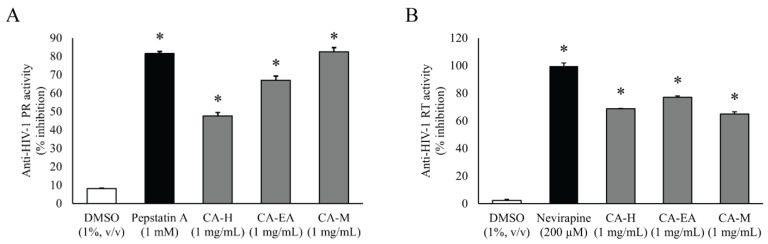
Inhibitory activities of CA extracts on HIV-1 PR (**A**) and HIV-1 RT (**B**). All extracts (1 mg/mL) significantly inhibited both HIV-1 PR and RT compared to DMSO control (One-way ANOVA, Dunnett’s test, * *p* < 0.05). Mean ± SD of at least three independent experiments is presented.

All CA extracts (1 mg/mL) significantly suppressed HIV-1 PR activity compared to DMSO (vehicle control). CA-M exhibited the highest inhibitory activity among all the extracts with the percentage of inhibition at 82.44 ± 2.33%. Interestingly, the percent inhibition of CA-M showed a similar result as that of the positive control Pepstatin A (81.48 ± 1.07%). Besides, CA-EA and CA-H showed inhibition at 67.05 ± 2.19 and 47.61 ± 1.77%, respectively, against HIV-1 PR (Figure 2A). Since, CA-M showed the best HIV-1 PR inhibitory activity, it was selected to test at multiple concentrations. The calculated half-maximal inhibitory concentration of CA-M on HIV-1 PR activity was 0.87 ± 0.02 mg/mL.

### 2.3. HIV-1 Reverse Transcriptase Inhibitor Screening

To screen in vitro HIV-1 RT inhibitory activity, the extracts were tested at the concentration of 1 mg/mL. All CA extracts showed significant inhibition against HIV-1 RT activity compared to DMSO. Among all extracts, CA-EA exhibited the highest HIV-1 RT inhibitory effect at 76.93%, followed by CA-H (68.82%) and CA-M (64.97%), respectively. The positive control, Nevirapine (200 µM), exhibited an inhibition of 99.66 ± 2.28% (Figure 2B).

### 2.4. Phytochemical Profile Analysis

**Figure 3:**
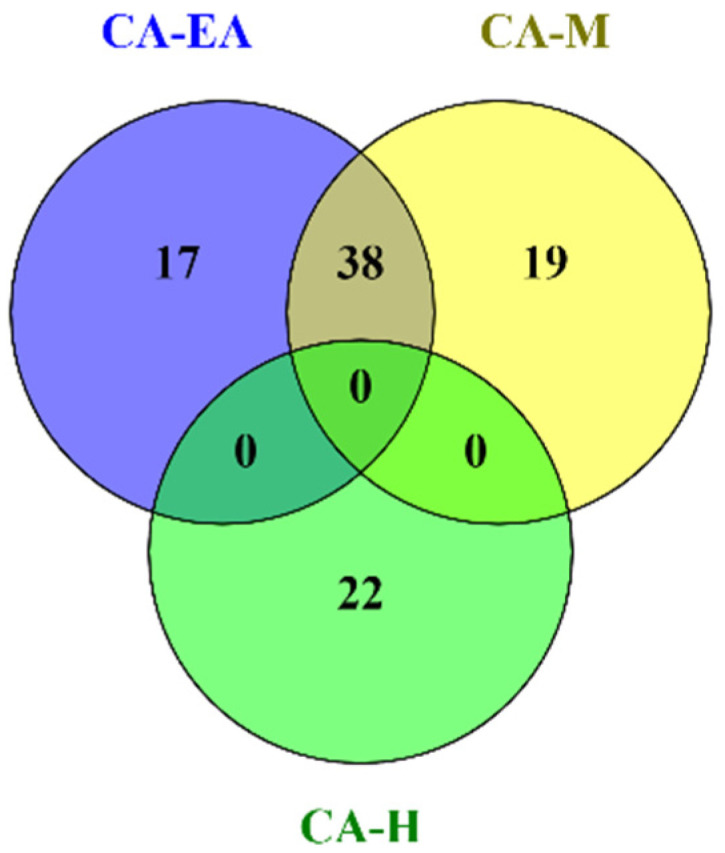
The Venn diagram shows numbers of compounds found in CA-H, CA-EA, and CA-M.

Phytochemical constituents in CA-H were analyzed and identified by using GC-MS after comparing the observed mass to the theoretical mass. The major compounds found in CA-H were terpenoids and fatty acids (Supplementary data S1). The chemical profiles of CA-EA and CA-M were investigated by using UPLC-HRMS. We found that CA-EA and CA-M were mainly composed of fatty acids, amino acids, and terpenoids (Supplementary data S2 and S3). As shown in Figure 3, the Venn diagram demonstrated that 38 compounds were detected in both CA-EA and CA-M. Besides, CA-H was composed of 22 unique compounds. All identified compounds found in CA extracts were tabulated in Table 1.

### 2.5. Evaluation of Lipinski′s Rule of Five and ADMET Parameters

**Figure 4:**
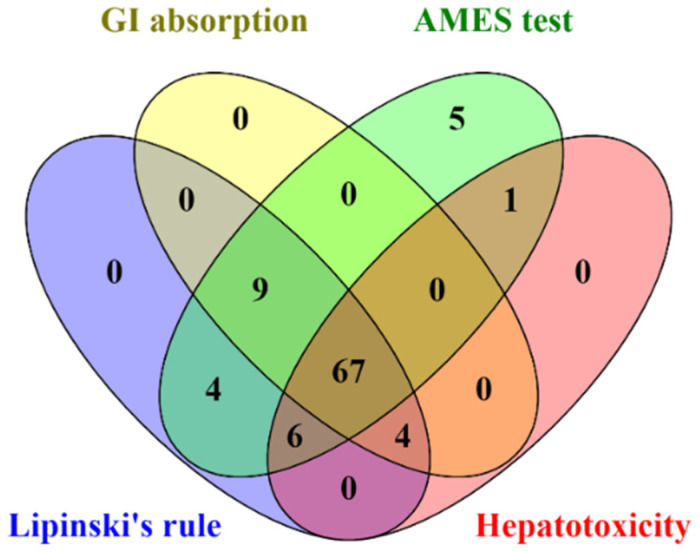
The Venn diagram presents the number of compounds predicted as high GI absorption, no AMES toxicity, no hepatotoxicity, and acceptable for Lipinski’s rule.

All identified compounds found in CA extracts were evaluated for their drug-likeness properties. The in silico results indicated that 67 out of 96 compounds complied with Lipinski’s rule and showed high GI absorption, with no AMES toxicity, and hepatotoxicity (Figure 4 and Supplementary data S4). Therefore, these 67 filtered compounds were further studied for their ability to inhibit HIV-1 PR and RT by molecular docking.

### 2.6. Prediction of Binding Affinity of Candidate Ligands against HIV-1 PR Active Site

To predict the ability of candidate compounds from CA extracts on HIV-1 PR blocking, in silico molecular docking study was performed. Initially, Amprenavir, an HIV-1 PR inhibitory drug, originally complexed with HIV-1 PR (PDB: 5KR0) was removed and re-docked into the original binding site using AutoDock 4.2. The predicted binding energy of Amprenavir (−9.73 kcal/mol) at HIV-1 PR active site was used as a benchmark for the comparison of other candidate compounds. Interestingly, 4 out of 67 compounds showed binding energies on the active site of HIV-1 PR lower than that of Amprenavir, as shown in Table 2 and Supplementary data S5. The following compounds, dihydroergocornine, 27-nor-5β-cholestane-3α,7α,12α,24,25-pentol, 3β,6α,7α-trihydroxy-5β-cholan-24-oic acid, and 6β,11β,16α,17α,21-pentahydroxypregna-1,4-diene-3,20-dione-16,17-acetonide provided binding energies of −12.65, −11.53, −10.92, and −10.71 kcal/mol, respectively.

**Figure 5:**
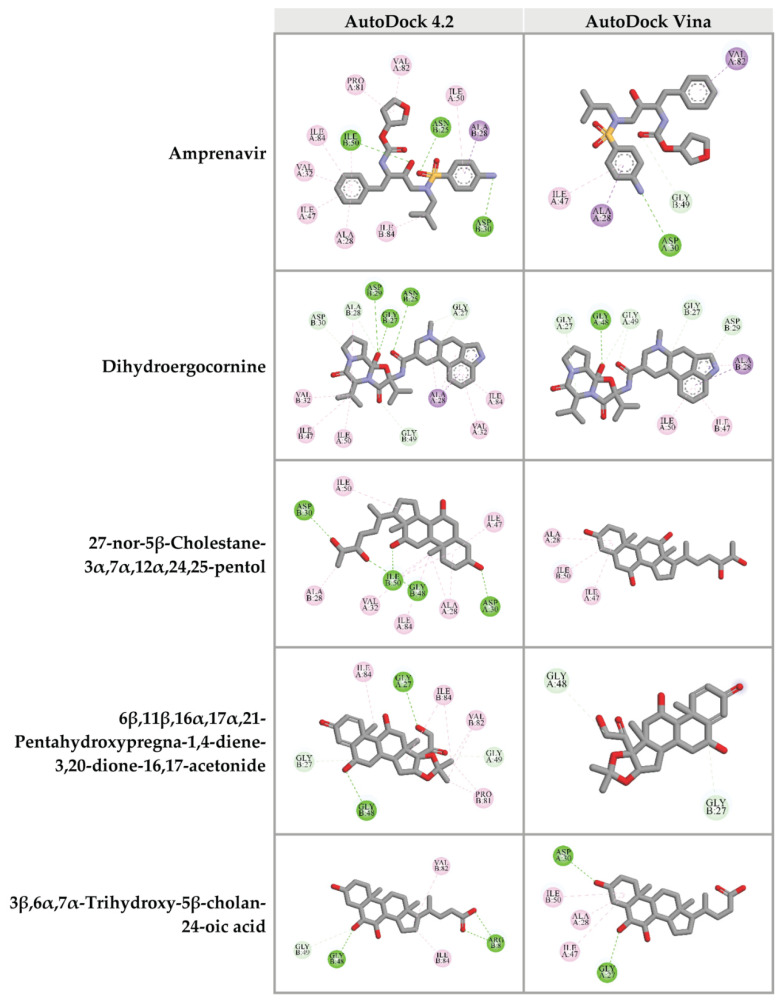
Three-dimensional schematic interaction between HIV-1 PR (PDB: 5KR0) and candidate ligands. Balls and sticks represent amino acid residues and ligands, respectively. Green dashed lines indicate hydrogen bonds. Pink and purple dashed lines indicate hydrophobic bonds.

To confirm the binding ability of top 10 candidates in Table 2, AutoDock Vina was used as another platform for docking against HIV-1 PR. Four ligands, dihydroergocornine, 27-nor-5β-cholestane-3α,7α,12α,24,25-pentol, 6β,11β,16α,17α,21-pentahydroxypregna-1,4-diene-3,20-dione-16,17-acetonide, and 3β,6α,7α-trihydroxy-5β-cholan-24-oic acid, exhibited lower binging energies (−11.3, −9.8, −9.7, −9.3 kcal/mol, respectively) against the active site of HIV-1 PR than that of Amprenavir (−9.0 kcal/mol). Interestingly, the results from both AutoDock 4.2 and AutoDock Vina programs showed a positive correlation. The schematic interactions between amino acid residues of HIV-1 PR and candidate ligands are presented in Figure 5. These findings indicated that the four candidate compounds found in CA-EA and CA-M extracts potentially served as HIV-1 PR inhibitors.

### 2.7. Prediction of Binding Affinity of Candidate Ligands against DNA Polymerase Active Site of HIV-1 RT

HIV-1 RT is another interesting drug target for HIV-1 life cycle inhibition. The HIV-1 RT is a multifunctional enzyme, including DNA polymerase and RNase H [5]. In this study, we focused on both active sites of HIV-1 RT. At the DNA polymerase domain, the crystal structure of HIV-1 RT co-crystallized with Nevirapine (PDB: 3QIP) was used to perform molecular docking. Nevirapine, an original inhibitor, was re-docked into the DNA polymerase site of HIV-1 RT to obtain reference values for the interpretation of data. The re-docking results showed that Nevirapine was fitted in the DNA polymerase active site with a binding energy of -9.31 kcal/mol. Interestingly, 7 out of 67 compounds from CA extracts provided binding energies to the DNA polymerase site less than that of Nevirapine, including 3β,6α,7α-trihydroxy-5β-cholan-24-oic acid (−10.39 kcal/mol); 27-nor-5β-cholestane-3α,7α,12α,24,25-pentol (−9.99 kcal/mol); xanthumin (−9.73 kcal/mol); prostaglandin F1a alcohol (−9.72 kcal/mol); QH2 (−9.69 kcal/mol); prostaglandin H1 (−9.50 kcal/mol); and lactone of PGF-MUM (-9.48 kcal/mol), as shown in Table 3 and Supplementary data S6.

**Figure 6:**
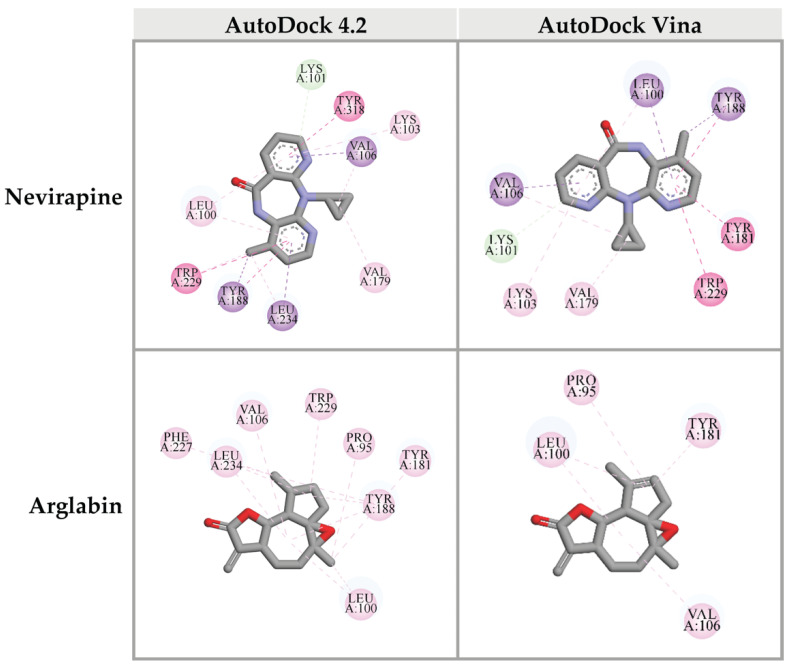
Three-dimensional schematic interaction between HIV-1 RT (PDB: 3QIP) at the DNA polymerase active site and candidate ligands. Balls and sticks represent amino acid residues and ligands, respectively. Green dashed lines indicate hydrogen bonds. Pink and purple dashed lines indicate hydrophobic bonds.

Top 10 candidate compounds were subsequently investigated for the binding affinity against the DNA polymerase site of HIV-1 RT using Autodock Vina program. Unfortunately, none of CA-identified compounds provided the binding affinity better than that of the reference control, Nevirapine (−11.7 kcal/mol). However, arglabin showed the lowest binding energy at −10.8 kcal/mol, among all the candidate ligands. Interactions between these ligands and HIV-1 RT were shown in Figure 6.

### 2.8. Prediction of Binding Affinity of Candidate Ligands against RNase H Active Site of HIV-1 RT

**Figure 7:**
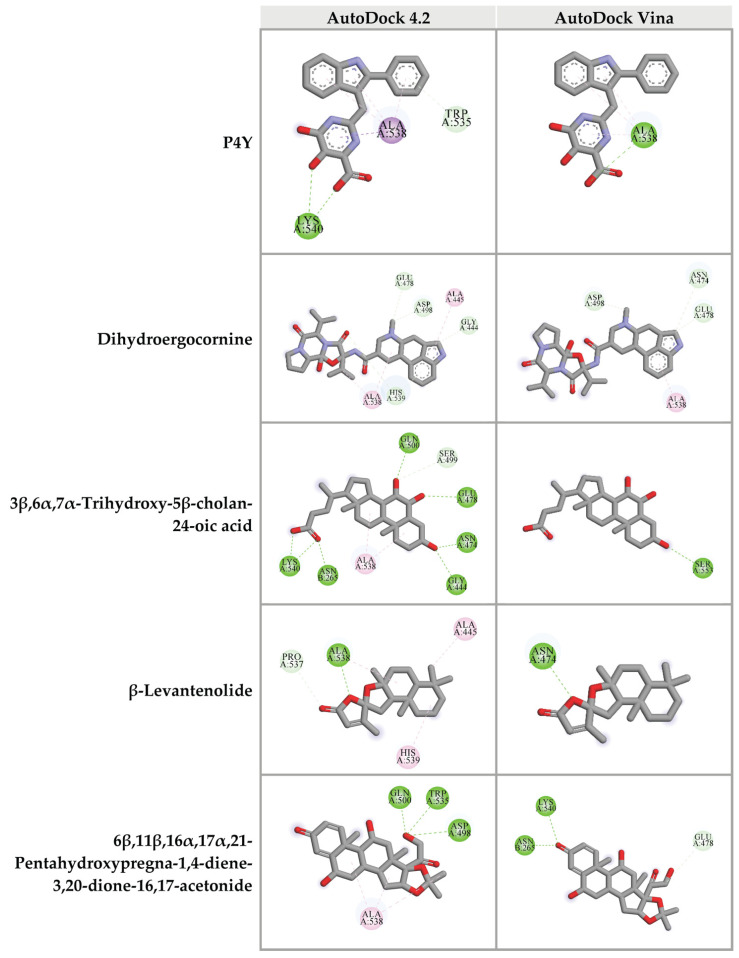
Three-dimensional schematic interaction between HIV-1 RT (PDB: 3QIP) at the RNase H active site and candidate ligands. Balls and sticks represent amino acid residues and ligands, respectively. Green dashed lines indicate hydrogen bonds. Pink and purple dashed lines indicate hydrophobic bonds.

For the RNase H domain of HIV-1 RT, P4Y was re-docked into the binding site, which was defined by the original binding site of the original RNase H inhibitor of HIV-1 RT (PDB: 3QIP). The binding energy between P4Y and RNase H domain exhibited at −5.29 kcal/mol. Among all candidate compounds from CA extracts, eight compounds exhibited lower binding energy against RNase H domain than that of the original inhibitor. 3β,6α,7α-trihydroxy-5β-cholan-24-oic acid provided the strongest binding affinity to RNase H active site with a binding energy of −6.77 kcal/mol, followed by 27-nor-5β-cholestane-3α,7α,12α,24,25-pentol (−6.58 kcal/mol); hydroxyibuprofen (−5.92 kcal/mol); 6β,11β,16α,17α,21-pentahydroxypregna-1,4-diene-3,20-dione-16,17-acetonide (−5.90 kcal/mol); dihydroergocornine (−5.80 kcal/mol); lactone of PGF-MUM (−5.57 kcal/mol); Pro Glu (−5.44 kcal/mol); β-levantenolide (−5.38 kcal/mol); and Val Val (−5.34 kcal/mol), respectively (Table 4 and Supplementary data S7). The ligand-receptor interactions demonstrated that these candidate ligands were docked in the same pocket as the reference inhibitor. Moreover, they interacted with some amino acid residues, which were found in the P4Y complex with HIV-1 RT (Figure 7).

Furthermore, top 10 candidates were confirmed for their biding affinity against RNase H active site of HIV-1 RT using AutoDock Vina. There were four compounds that exhibited the binding energy lower than that of the original inhibitor P4Y (−6.0 kcal/mol). The following compounds, dihydroergocornine, 27-nor-5β-cholestane-3α,7α,12α,24,25-pentol, 3β,6α,7α-trihydroxy-5β-cholan-24-oic acid, β-levantenolide, and 6β,11β,16α,17α,21-pentahydroxypregna-1,4-diene-3,20-dione-16,17-acetonide showed the binding affinity of −6.5, −6.4, −6.4, and −6.3 kcal/mol, respectively. It is interesting that four ligands also showed lower binding energy than P4Y via AutoDock 4.2. Our results indicated that these four candidate ligands found in CA-EA and CA-M extracts potentially inhibit HIV-1 RT at the RNase active site.

### 2.9. Investigations of Antioxidant Constituents and Antioxidant Activity

**Figure 8:**
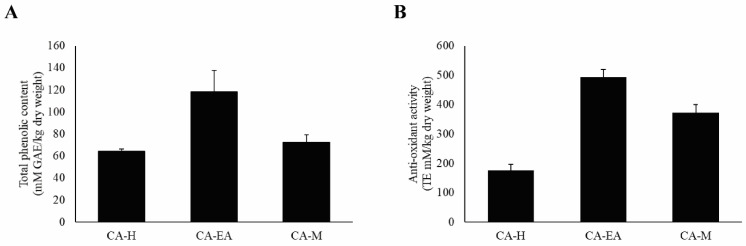
Analysis of antioxidant compounds and antioxidant capacity of CA extracts. (**A**) Total phenolic content of CA extract. (**B**) Antioxidant activity of CA extracts. Data are shown as mean ± SD of at least three independent experiments.

According to previous study, HIV-1 infection can cause oxidative stress accumulation in the infected cells, leading to immune cell death [22]. Therefore, we would like to evaluate both the antioxidant constituents, including phenols by Folin-Ciocalteu’s assay and the antioxidant activity by Trolox equivalent antioxidant capacity (TEAC) assay. For total phenol investigation, the results showed that CA-EA had the highest phenolic content at 118.38 ± 19.53 mM of gallic acid equivalent (GAE)/kg dry weight, followed by CA-M (72.39 ± 7.06 mM GAE/kg dry weight) and CA-H (64.16 ± 2.32 mM GAE/kg dry weight), respectively (Figure 8A).

Besides, the antioxidant capacity of all CA extracts was determined by measuring ABTS scavenging activity. CA-EA exhibited the highest antioxidant activity with a value of 494.68 ± 24.58 TE mM/kg dry weight, followed by CA-M (371.55 ± 28.86 TE mM/kg dry weight) and CA-H (176.52 ± 20.47 TE mM/kg dry weight) (Figure 8B). Interestingly, the observed activity indicated that the more total phenolic content provided the higher antioxidant activity of CA extracts.

## 3. Discussion

Although HIV-1 infection has been found for many years, this infection is still uncurable due to the complexity of the virus. Patients who are infected with HIV-1 cannot eradicate the viruses from their bodies and need to control the number of viruses by continuously taking anti-retroviral drugs. Now, many effective antiviral drugs are available but the side effects from using these drugs are also found. The typical adverse effects of antiretroviral drugs are hepatotoxicity [23], mitochondrial toxicity [6], hypersensitivity, and lipodystrophy [24]. The development of highly effective drugs with low adverse effects is a critical study for the better life of HIV-1 infected patients. Natural products have been investigated for their pharmaceutical activities and used as a scaffold for novel drug development [25,26].

Herein, we focused on inhibitory effects of CA extracts and their derived compounds against HIV-1 enzymes: PR and RT. Our results showed that CA extracts served as potential HIV-1 inhibitors. The in vitro screening assays demonstrated that all CA extracts (1 mg/mL) showed significant inhibition against both enzyme activities. Among all extracts, CA-M showed the strongest inhibition against HIV-1 PR activity. Interestingly, the effect of CA-M on HIV-1 PR inhibition was comparable to the effect of Pepstatin A (1 mM). A previous study on HIV-1 PR inhibitory activity, *Alpinia galanga*, a plant in Zingiberaceae, displayed that the methanol extract inhibited HIV-1 PR more effectively than the aqueous extract [27]. Yu YB et al. demonstrated that alnustic acid methyl ester, a triterpenoid isolated from methanol extract of *Alnus firma* could exhibit inhibition against HIV-1 PR [28]. Hydroxypanduratin A and panduratin A isolated from methanol extract of *Boesenbergia pandurata* possessed anti-HIV-1 PR activity [29]. These data indicate that the methanol extract of CA rhizomes might composed of phytochemical active compound(s) that potentially inhibit HIV-1 PR activity.

The hallmark characteristic of HIV-1 infection is the depletion of immune cells, especially CD4^+^ T-cells. A previous study demonstrated that the decrease of the CD4^+^ T-cells was triggered by the HIV-1 envelope glycoproteins, leading to oxidative stress accumulation and cell death [22]. Treatment of antioxidative agent, N-acetylcysteine, in the HIV-1 infected patients could up-regulate glutathione level and total antioxidant status, whereas lipid peroxidation was reduced. [30]. These data suggest that the control of HIV-1 load and antioxidant status are important keys for the treatment of people who live with HIV-1. Our current results showed that CA extracts exhibited not only anti-HIV-1 but also antioxidant activities. Moon-ai W and colleagues reported that crude homogenate and ammonium sulfate cut fraction of CA rhizomes contained high superoxide dismutase activity which indicated the antioxidative effect [31]. Moreover, Zingiberaceae plants such as *Curcuma longa*, *Zingiber officinale*, and *Kaempferia parviflora* exhibited antioxidant activity by scavenging 1,1-diphenyl-2-picrylhydrazyl (DPPH) and 2,20-azino-bis(3-ethylbenzothiazoline-6-sulfonic acid) diammonium salt (ABTS) free radicals [32,33].

Phytochemical constituents in CA extracts were analyzed using GC-MS and UPLC-HRMS techniques. The compounds in CA-H extract were identified by GC-MS; terpenoids and fatty acids were mostly found in this fraction. In addition, CA-EA and CA-M extracts were analyzed by UPLC-HRMS. These extracts were mainly composed of fatty acids, amino acids, and terpenoids. A previous study reported the presence of a high amount of sugars, organic acids, alkanes, fatty acids, and terpenoids in methyl tert-butyl ether, methanol, and chloroform extracts of CA rhizomes [34]. Similarly, our finding discovered α-terpineol, oleic acid, palmitic acid, and linoleic acid as in the previous study [34]. Interestingly, our previous study reported that linoleic acid isolated from *Auricularia polytricha*, an edible mushroom, could inhibit HIV-1 PR activity [12].

The drug-likeness property of identified compounds from CA extracts was considered based on Lipinski’s rule of five [35,36], absorption property, and toxicity [37]. Sixty-seven compounds showed the possibility to be drugs with high absorption and no toxicity. Furthermore, in silico molecular docking was performed to predict the ability of candidate compounds in both HIV-1 PR and RT inhibitions. Based on the binding energy values, there are 4 candidate compounds: dihydroergocornine; 27-nor-5β-cholestane-3α,7α,12α,24,25-pentol; 3β,6α,7α-trihydroxy-5β-cholan-24-oic acid; and 6β,11β,16α,17α,21-pentahydroxypregna-1,4-diene-3,20-dione-16,17-acetonide could bind to the active residue of HIV-1 PR stronger than that of Amprenavir, a benchmark ligand. As shown in Table 1 and Table 2, dihydroergocornine, which provided the highest binding affinity was detected in CA-M. The following three compounds, 27-nor-5β-cholestane-3α,7α,12α,24,25-pentol, 3β,6α,7α-trihydroxy-5β-cholan-24-oic acid, and 6β,11β,16α,17α,21-pentahydroxypregna-1,4-diene-3,20-dione-16,17-acetonide, were found in both CA-EA and CA-M. However, none of the identified compounds in CA-H showed strong binding affinity to the HIV-1 PR active site. These results supported the in vitro findings that CA-M exhibited the highest HIV-1 PR inhibition, followed by CA-EA and CA-H, respectively.

Focusing on the chemical structures (Figure 6), the following compounds, 27-nor-5β-cholestane-3α,7α,12α,24,25-pentol, 3β,6α,7α-trihydroxy-5β-cholan-24-oic acid, and 6β,11β,16α,17α,21-pentahydroxypregna-1,4-diene-3,20-dione-16,17-acetonide were characterized as triterpenoids with similar steroid structure. Previous studies also found that triterpenoids isolated from natural sources could inhibit HIV-1 PR activity [28,38,39]. Ergosterol, a mushroom triterpenoid isolated from *Auricularia polytricha*, suppressed HIV-1 PR activity [12]. We suggest that plant-derived triterpenoids and their derivatives are attractive compounds for anti-HIV-1 PR drug development. The triterpenoid-rich plants could be focused as sources of traditional or supplementary importance for anti-viral herbal medicines.

HIV-1 RT plays multifunctional activities, including DNA polymerase and RNase H activities. The DNA polymerase generates cDNA from viral RNA template while the RNase H degrades viral RNA. The two enzymatic activities of HIV-1 RT cooperate to convert the viral RNA into cDNA in the reverse transcription step of the HIV-1 replication cycle [5]. Therefore, interferences of these HIV-1 RT active sites can block the viral replication process. In our current study, we evaluated the possibility of identified compounds from CA extracts on inhibition of both DNA polymerase and RNase H activities. Computer-aid molecular docking by AutoDock 4.2 software demonstrated that 3β,6α,7α-trihydroxy-5β-cholan-24-oic acid; 27-nor-5β-cholestane-3α,7α,12α,24,25-pentol; xanthumin; prostaglandin F1a alcohol; QH2; prostaglandin H1; and lactone of PGF-MUM potentially bound DNA polymerase domain of HIV-1 RT. On the contrary, the results from AutoDock Vina exhibited that none of candidates provided the better binding affinity than the reference molecule, as shown in Table 3. The different algorithms of AutoDock 4.2 and AutoDock Vina may cause the different predicted binding energies [40,41]. AutoDock Vina is an improved accuracy of molecular docking program developed from AutoDock 4.2 [42]. Nevertheless, arglabin and Nevirapine showed almost the same binding energy value. Interestingly, the docking results of arglabin were relevant in both molecular docking platforms. Besides, these results supported our in vitro screening for polymerase activity of HIV-1 RT inhibition by CA extract. We also found that CA extracts moderately suppressed HIV-RT activity (Figure 2B).

At RNase H active site, these compounds, dihydroergocornine, 3β,6α,7α-trihydroxy-5β-cholan-24-oic acid, β-levantenolide, and 6β,11β,16α,17α,21-pentahydroxypregna-1,4-diene-3,20-dione-16,17-acetonide provided strong binding affinity via both AutoDock 4.2 and AutoDock Vina programs. These compounds were found in CA-H, CA-EA, and CA-M. These findings supported the in vitro results that all CA extracts showed no significant difference of the percent inhibition on HIV-1 RT activity.

Interestingly, dihydroergocornine, 3β,6α,7α-trihydroxy-5β-cholan-24-oic acid, and 6β,11β,16α,17α,21-pentahydroxypregna-1,4-diene-3,20-dione-16,17-acetonide exhibited high binding affinity to both HIV-1 PR and RT (RNase H site). These results suggest that these three compounds potentially serve as HIV-1 PR and RT inhibitors. Dihydroergocornine is an approved drug for hypotensive treatment which is derived from ergocornine, an alkaloid isolated from crude ergot extract [43]. A previous report demonstrated that methanolic extract of *Acalypha indica* leaves which is composed of 27-nor-5β-cholestane-3α,7α,12α,24,25-pentol and 3β,6α,7α-trihydroxy-5β-cholan-24-oic acid exhibited antioxidative effect [44]. Based on our findings, these two antioxidative compounds were also found in both CA-EA and CA-M. The two extracts exhibited higher antioxidant activity compared to CA-H. These results suggest that 27-nor-5β-cholestane-3α,7α,12α,24,25-pentol and 3β,6α,7α-trihydroxy-5β-cholan-24-oic acid might be the free radical scavengers in CA-EA and CA-M that provided the observed antioxidation. Moreover, 3β,6α,7α-trihydroxy-5β-cholan-24-oic acid was also found in the root and rhizome extract of *Canna indica* L. [45] and exhibited potent activity against enterohepatic disorders [46]. However, the effects of these compounds on HIV-PR and RT inhibitions have never been investigated. Our study was the first to report the anti-HIV-1 PR and RT activities of the compounds.

This study provided the promising active compounds that might inhibit HIV-1 PR and RT activities. These data will be an interesting idea for anti-HIV drug development from plant-based sources. Moreover, this zingiber is commonly used in Eastern traditional medicine, knowing of novel medicinal property is a value-added of this herb. Besides, other herbs that contain these ingredients may also provide the similar biological properties, especially the plants in Zingiberaceae family. Furthermore, the effects of individual compounds on HIV-1 PR and RT inhibitions remain to be elucidated.

To summarize, methanol extract of CA exhibited strong anti-HIV-1 PR and mild inhibition of HIV-1 RT. Moreover, we reported the possible active components in the CA extract that might have anti-HIV-1 PR and RT activities. We suggest that CA could be a beneficial source of natural products with anti-retroviral properties against HIV-1. Furthermore, the determination of anti-HIV-1 effects of the proposed compounds should be additionally studied under suitable in vitro/in vivo conditions.

## 4. Materials and Methods

### 4.1. Plant Material and Extract Preparation

The rhizomes of *Curcuma aeruginosa* Roxb. (CA) were collected from the Chatuchak market, Bangkok, Thailand (13.7995° N, 100.5492° E). The rhizomes were dried and blended into powders. The powders were extracted using the maceration technique. Firstly, the plant material (200 g) was macerated in methanol (500 mL) and incubated for 72 h. Subsequently, the methanol extract was collected, filtered, and then evaporated using a rotary evaporator to get crude extract (12.14 g). After that, the crude extract was fractionated by hexane (200 mL). The hexane solution was collected and evaporated to obtain a hexane extract (CA-H, 0.22 g). Next, the residue from hexane extraction was fractionated by ethyl acetate (200 mL). The ethyl acetate soluble liquor was collected and concentrated by rotary evaporation to make ethyl acetate extract (CA-EA, 5.18 g). Finally, the residue from ethyl acetate extraction was extracted with methanol (100 mL) to obtain a methanol extract (CA-M, 1.24 g). The schematic of extraction is shown in Figure 1.

### 4.2. HIV-1 Protease Inhibitor Screening

HIV-1 protease inhibitory activity of the sample (1 mg/mL) was measured using a fluorometric HIV-1 protease inhibitor screening kit (Biovision Inc. USA) according to the manufacturer’s recommendation. One millimolar of Pepstatin A and 1% (*v/v*) of DMSO were utilized as a known HIV-1 PR inhibitor and vehicle control, respectively. The fluorescence was measured in a kinetic mode 330 (excitation) and 450 nm (emission) for 90 min at 37 °C using the PerkinElmer EnSpire plate reader (Perkin-Elmer).

### 4.3. HIV-1 Reverse Transcriptase Inhibitor Screening

The extracts at 1 mg/mL were tested for HIV-1 RT inhibitor using an HIV-1 reverse transcriptase assay, colorimetric kit (Roche, Germany) according to manufacturer’s protocol. Nevirapine (200 μM), a HIV-1 RT inhibitory drug was used as a positive control. DMSO (1%, *v/v*) was determined as a vehicle control. The color reactions were measured at wavelength 405 nm and 490 nm as a reference wavelength by using an EnSpire plate reader (Perkin-Elmer).

### 4.4. Phytochemical Profile Analysis

The CA-H extract was subjected to Gas Chromatography-Mass Spectrometry (GC-MS) analysis for the identification of phytochemical profile. While CA-EA and CA-M extracts were analyzed for their chemical profiles by Ultra Performance Liquid Chromatography-High Resolution Mass Spectroscopy (UPLC-HRMS, Agilent Technologies, Santa Clara, CA, USA) analysis (Institute of Chemical Technology, India). The compounds were identified by comparing an observed mass to the theoretical mass available in the public database.

### 4.5. Evaluation of Lipinski’s Rule of Five and ADMET Parameters

To determine drug-likeness prediction, Lipinski’s rule of five and absorption property of the phytochemicals were evaluated using the SwissADME online program (http://www.swissadme.ch, accessed on 17 August 2021 [36]. Besides, toxicity parameters were predicted by submitting the SMILES files of the small molecules to the pkCSM online database (http://biosig.unimelb.edu.au/pkcsm/prediction, accessed on 17 August 2021 [47]. The compounds that passed Lipinski’s rule and were predicted to have high gastrointestinal (GI) absorption and nontoxicity were further studied for in silico molecular docking.

### 4.6. Molecular Docking Analysis

HIV-1 protease (PDB: 5KR0) [48] and HIV-1 RT (PDB: 3QIP) [49] were obtained from the Protein Data Bank. The proteins were prepared using AutoDockTools-1.5.6. The water molecules and the original ligands: Amprenavir, Nevirapine, and 5,6-dihydroxy-2-[(2-phenyl-1H-indol-3-yl) methyl] pyrimidine-4-carboxylic acid (P4Y) were removed. Moreover, all missing hydrogen atoms were added in the protein structures. Then the file format was converted to PDBQT before molecular docking analysis. Energy minimization for the ligands was done using BIOVIA Discovery Studio 2020 software. All candidate ligands were docked against HIV-1 PR and RT by using AutoDock 4.2 [13]. For HIV-1 PR, the grid box was set to 60 × 60 × 60 xyz points of size and 0.375 Å of spacing at the center of HIV-1 PR. Moreover, two active sites of HIV-1 RT, including polymerase and RNase H active sites, were studied. The parameters of grid boxes for polymerase and RNase H active sites were set to 40 × 40 × 40 xyz points of size, 0.375 Å of spacing, and grid center at 9.831 × 13.983 × 17.1 xyz; and 35 × 35 × 35 xyz points of size, 0.375 Å of spacing, and grid center at 0.144 × 74.854 × 34.626 xyz, respectively. The top 10 compounds provided the highest binding affinity were further run on another molecular docking platform, AutoDock Vina program [42] to support the AutoDock 4.2 results. Hydrogen bond interactions of the docked structures were further analyzed using the Discovery Studio Visualizer.

### 4.7. Total Phenolic Content Measurement

The total phenolic content in the extracts was assessed by Folin-Ciocalteu’s method [50]. Briefly, the extract at 1 mg/mL was mixed with the Folin-Ciocalteu’s phenol reagent (10%, *v/v*) in a 96-well plate, followed by incubation in dark for 20 min. Next, 7.5% (*w/v*) of sodium carbonate (Na_2_CO_3_) was added and further incubated for 20 min. The absorbance at 760 nm was measured by using a microplate reader (BioTek Synergy Mx). Gallic acid was employed as the standard compound. The results were represented as mM of gallic acid equivalent (GAE)/kg dry weight.

### 4.8. Antioxidant Activity Measurement

The antioxidant activity of the extracts was detected by Trolox equivalent antioxidant capacity (TEAC) assay, following the previous study of Panthong S et al. [51]. All CA extracts (1 mg/mL) were determined for ABTS radical scavenging capacity comparing to Trolox, a vitamin E analogue. The results were reported in Trolox equivalent (TE) mM/kg dry weight.

### 4.9. Statistical Analysis

All values were expressed as the mean ± standard deviation (SD) of triplicated experimental analysis. Statistical significance was analyzed by one-way ANOVA, Dunnett’s test using SPSS 16.0 software. *p* < 0.05 was considered significant.

## 5. Conclusions

Our current study demonstrated a novel biological activity of *Curcuma aeruginosa* Roxb. (CA) extracts on HIV-1 inhibition by suppressing HIV-RT and HIV-1 PR activities. In vitro screenings of CA extracts (1 mg/mL) showed that all CA extracts exhibited moderate inhibition against HIV-1 RT activity ranging from 64.97 to 76.93%. Interestingly, the methanolic fraction (CA-M) provided the highest HIV-1 PR inhibitory activity at 82.44%. Besides, CA-EA exhibited the highest antioxidant effect, followed by CA-M and CA-H, respectively. The compositions of phenolic compounds present in CA extracts were related to the radical scavenging activity of the extracts. Furthermore, phytochemical profiling showed that CA extracts were mainly composed of fatty acids, amino acids, and terpenoids. Additionally, in silico virtual screening indicated that dihydroergocornine, 3β,6α,7α-trihydroxy-5β-cholan-24-oic acid, and 6β,11β,16α,17α,21-Pentahydroxypregna-1,4-diene-3,20-dione-16,17-acetonide provided dual enzymatic inhibition on HIV-1 PR and RT. This study obtained beneficial data for anti-HIV-1 drug development from *C. aeruginosa* Roxb. extracts.

## Figures and Tables

**Table 1:** List of phytochemical compounds identified in CA extracts.

Identified Compound	Extract
(22S)-1α,25-Dihydroxy-22-methoxy-26,27-dimethyl-23,24-tetradehydro-20-epivitamin D3	CA-EA
(4Z)-4-(6,6-Dimethyl-2-methylidenecyclohex-3-en-1-ylidene) pentan-2-ol	CA-H
(6R)-Vitamin D3 6,19-(4-phenyl-1,2,4-triazoline-3,5-dione) adduct/(6R)-cholecalciferol 6,19-(4-phenyl-1,2,4-triazoline-3,5-dione) adduct	CA-M
(E)-2-Methylglutaconic acid	CA-EA, CA-M
10-keto Tridecanoic acid	CA-EA
12-Hydroxy-10-octadecynoic acid	CA-EA, CA-M
13-Hydroxy-tridecanoic acid	CA-M
1α-Hydroxy-24-(dimethylphosphoryl)-25,26,27-trinorvitamin D3	CA-M
2,2,4-Trimethyl-3-[(3E,7E,11E)-3,8,12,16-tetramethylheptadeca-3,7,11,15-tetraenyl] cyclohexan-1-ol	CA-H
2,4-Dimethyl-2-eicosenoic acid	CA-EA
2-[3-Carboxy-3-(methylammonio)propyl]-L-histidine	CA-EA, CA-M
27-nor-5β-Cholestane-3α,7α,12α,24,25-pentol	CA-EA, CA-M
2-Hydroxyethanesulfonate	CA-EA
2-oxo-Dodecanoic acid	CA-EA
3-(3,3,8,8-Tetramethyl-5-tricyclo [5.1.0.02,5] oct-5-enyl) propanoic acid	CA-H
3-Dodecynoic acid	CA-M
3-n-Decyl acrylic acid	CA-EA, CA-M
3-oxo-Tridecanoic acid	CA-EA, CA-M
3-Tridecynoic acid	CA-M
3β,6α,7α -Trihydroxy-5β-cholan-24-oic acid	CA-EA, CA-M
4-(2-Hydroxy-3isopropylaminoproxy) benzyloxy acetic acid	CA-EA
4-(3,3-dimethylbut-1-ynyl)-4-hydroxy-2,6,6-trimethylcyclohex-2-en-1-one	CA-H
4,7,7-Trimethyl-4-(2-methylallyl) tricyclo [3.3.0.02,8] octane-3,6-dione	CA-H
4-Heptanone	CA-EA, CA-M
4-Hydroxy capric acid	CA-EA
4-Methylpentanal	CA-EA, CA-M
4Z-Decenedioic acid	CA-EA
6-(3-Hydroxyprop-1-en-2-yl)-4,8a-dimethyl-1,3,5,6,7,8-hexahydronaphthalen-2-one	CA-H
6E-Nonenoic acid	CA-EA, CA-M
6β,11β,16α,17α,21-Pentahydroxypregna-1,4-diene-3,20-dione-16,17-acetonide	CA-EA, CA-M
7E,9Z-Dodecadien-1-ol	CA-EA
7-Hydroxymethotrexate	CA-EA, CA-M
9-Dodecen-1-ol	CA-EA, CA-M
9-Isopropyl-1-methyl-2-methylene-5-oxatricyclo [5.4.0.03,8] undecane	CA-H
Ala Glu His	CA-M
Amiloxate	CA-EA, CA-M
Arglabin	CA-H
Benzenehexanoic acid, 2,5-dihydroxy-3,4-dimethoxy-6-methyl-	CA-EA, CA-M
Betaine	CA-M
Cadinol T	CA-H
Citronellic acid	CA-EA, CA-M
Cycloisolongifolene,8,9-dehydro-9-formyl-	CA-H
Cyclopropanebutanoic acid, 2-[[2-[[2-[(2-pentylcyclopropyl) methyl] cyclopropyl] methyl]	CA-H
Deoxyribose	CA-EA, CA-M
Deoxysappanone B 7,3′- dimethyl ether acetate	CA-EA
Dihydrocostunolide	CA-H
Dihydroergocornine	CA-M
Dihydrojasmonic acid, methyl ester	CA-EA, CA-M
Dihydrosphingosine	CA-EA, CA-M
Elephantopin	CA-M
Ethyl Oxalacetate	CA-M
Gemfibrozil	CA-EA, CA-M
Gemfibrozil M1	CA-EA, CA-M
Gemfibrozil M3	CA-EA, CA-M
Gln Lys Arg	CA-EA, CA-M
GPEtn(12:0/0:0)	CA-EA, CA-M
Hexadecasphinganine	CA-EA, CA-M
Hydroxycyclohexanecarboxylic acid	CA-EA, CA-M
Hydroxyibuprofen	CA-EA, CA-M
Ibutilide	CA-M
Ile Asp	CA-EA
Ile Leu Leu	CA-EA, CA-M
Ile Thr	CA-EA
Isoaromadendrene epoxide	CA-H
Lactone of PGF-MUM	CA-EA, CA-M
Leucine	CA-M
Linoleic acid	CA-H
Linoleic acid, methyl ester	CA-H
Methyl jasmonate	CA-EA, CA-M
Methyldopexamine sulfate	CA-M
N-(2-fluro-ethyl)-eicosanoyl amine	CA-M
N-(2-hydroxyethyl) icosanamide	CA-M
Octanal	CA-EA, CA-M
Oleic Acid	CA-H
Palmitic acid	CA-H
Pantoic acid	CA-EA
Phe Ala Arg	CA-M
Phe Ala Pro	CA-EA
Phe Gln Arg	CA-EA, CA-M
Phytosphingosine	CA-EA, CA-M
Pro Glu	CA-EA
Prostaglandin F1a alcohol	CA-M
Prostaglandin H1	CA-EA, CA-M
Punctaporin B	CA-EA, CA-M
QH2	CA-EA, CA-M
Swietenine	CA-M
Taurine	CA-EA, CA-M
Trp Gln Trp	CA-EA
Undecanal	CA-EA
Val Glu	CA-EA, CA-M
Val Val	CA-M
Xanthumin	CA-H
α-Cadinol	CA-H
α-Terpineol	CA-H
β-Elemene	CA-H
β-Levantenolide	CA-H

**Table 2:** Top 10 molecular docking results of CA-identified compounds at the active site of HIV-1 PR.

Compound	Binding Energy (kcal/mol)	
	AutoDock 4.2	AutoDock Vina
Amprenavir (original inhibitor)	−9.73	−9.0
Dihydroergocornine	−12.65	−11.3
27-nor-5β-Cholestane-3α,7α,12α,24,25-pentol	−11.53	−9.8
3β,6α,7α-Trihydroxy-5β-cholan-24-oic acid	−10.92	−9.3
6β,11β,16α,17α,21-Pentahydroxypregna-1,4-diene-3,20-dione-16,17-acetonide	−10.71	−9.7
β-Levantenolide	−9.52	−8.2
Deoxysappanone B 7,3′-dimethyl ether acetate	−7.96	−7.8
Xanthumin	−7.84	−7.4
Punctaporin B	−7.67	−7.1
Dihydrocostunolide	−7.51	−7.6
Prostaglandin H1	−7.45	−6.4

**Table 3:** Top 10 molecular docking results of CA-identified compounds at the DNA polymerase active site of HIV-1 RT.

Compound	Binding Energy (kcal/mol)	
	AutoDock 4.2	AutoDock Vina
Nevirapine (original inhibitor)	−9.31	−11.7
3β,6α,7α-Trihydroxy-5β-cholan-24-oic acid	−10.39	−5.4
27-nor-5β-Cholestane-3α,7α,12α,24,25-pentol	−9.99	−2.8
Xanthumin	−9.73	−8.9
Prostaglandin F1a alcohol	−9.72	−8.4
QH2	−9.69	−8.6
Prostaglandin H1	−9.50	−7.9
Lactone of PGF-MUM	−9.48	−8.7
Dihydrocostunolide	−9.24	−9.9
Cadinol T	−9.22	−9.7
Arglabin	−9.22	−10.8

**Table 4:** Top 10 molecular docking results of CA-identified compounds at the RNase H active site of HIV-1 RT.

Compound	Binding Energy (kcal/mol)	
	AutoDock 4.2	AutoDock Vina
P4Y (original inhibitor)	−5.29	−6.0
3β,6α,7α-Trihydroxy-5β-cholan-24-oic acid	−6.77	−6.4
27-nor-5β-Cholestane-3α,7α,12α,24,25-pentol	−6.58	−5.9
Hydroxyibuprofen	−5.92	−4.3
6β,11β,16α,17α,21-Pentahydroxypregna-1,4-diene-3,20-dione-16,17-acetonide	−5.90	−6.3
Dihydroergocornine	−5.80	−6.5
Lactone of PGF-MUM	−5.57	−4.7
Pro Glu	−5.44	−4.2
β-Levantenolide	−5.38	−6.4
Val Val	−5.34	−4.1
6-(3-Hydroxyprop-1-en-2-yl)-4,8a-dimethyl-1,3,5,6,7,8-hexahydronaphthalen-2-one	−5.25	−4.9

## Data Availability

Data is contained within the article or supplementary material.

## Supplementary Materials

The following are available online at https://www.mdpi.com/article/10.3390/ph14111115/s1, Figure S1: Chromatograms of chemical constituents presented in CA-H, Table S1: Phytochemical constituents in CA-H identified by GC-MS, Figure S2: Chromatograms of chemical constituents presented in CA-EA, Table S2: Phytochemical constituents in CA-EA identified by UHPLC-HRMS, Figure S3: Chromatograms of chemical constituents presented in CA-M, Table S3: Phytochemical constituents in CA-M identified by UHPLC-HRMS, Table S4: Prediction of Lipinski’s rule of five, GI absorption, and toxicity parameters of identified compounds from CA extracts, Table S5: Molecular docking results of CA-identified compounds at the active site of HIV-1 PR, Table S6: Molecular docking results of CA-identified compounds at the DNA polymerase active site of HIV-1 RT, Table S7: Molecular docking results of CA-identified compounds at the RNase H active site of HIV-1 RT.

## References

[1] Nyamweya S., Hegedus A., Jaye A., Rowland-Jones S., Flanagan K.L., Macallan D.C. (2013). Comparing HIV-1 and HIV-2 infection: Lessons for viral immunopathogenesis. Rev. Med. Virol..

[2] Kwan C.K., Ernst J.D. (2011). HIV and tuberculosis: A deadly human syndemic. Clin. Microbiol. Rev..

[3] Engelman A., Cherepanov P. (2012). The structural biology of HIV-1: Mechanistic and therapeutic insights. Nat. Rev. Microbiol..

[4] Lv Z., Chu Y., Wang Y. (2015). HIV protease inhibitors: A review of molecular selectivity and toxicity. HIV/AIDS.

[5] Sarafianos S.G., Marchand B., Das K., Himmel D.M., Parniak M.A., Hughes S.H., Arnold E. (2009). Structure and function of HIV-1 reverse transcriptase: Molecular mechanisms of polymerization and inhibition. J. Mol. Biol..

[6] Brinkman K., ter Hofstede H.J., Burger D.M., Smeitink J.A., Koopmans P.P. (1998). Adverse effects of reverse transcriptase inhibitors: Mitochondrial toxicity as common pathway. Aids.

[7] Ammassari A., Murri R., Pezzotti P., Trotta M.P., Ravasio L., De Longis P., Caputo L., Narciso P., Pauluzzi S., Carosi G. (2001). Self-Reported symptoms and medication side effects influence adherence to highly active antiretroviral therapy in persons with HIV infection. J. Acquir. Immune Defic. Syndr..

[8] Montessori V., Press N., Harris M., Akagi L., Montaner J.S. (2004). Adverse effects of antiretroviral therapy for HIV infection. Cmaj.

[9] Yeni P. (2006). Update on HAART in HIV. J. Hepatol..

[10] Palella F.J., Delaney K.M., Moorman A.C., Loveless M.O., Fuhrer J., Satten G.A., Aschman D.J., Holmberg S.D., Investigators H.O.S. (1998). Declining morbidity and mortality among patients with advanced human immunodeficiency virus infection. N. Engl. J. Med..

[11] Esposito F., Corona A., Tramontano E. (2012). HIV-1 reverse transcriptase still remains a new drug target: Structure, function, classical inhibitors, and new inhibitors with innovative mechanisms of actions. Mol. Biol. Int..

[12] Sillapachaiyaporn C., Nilkhet S., Ung A.T., Chuchawankul S. (2019). Anti-HIV-1 protease activity of the crude extracts and isolated compounds from Auricularia polytricha. BMC Complementary Altern. Med..

[13] Sillapachaiyaporn C., Chuchawankul S. (2020). HIV-1 protease and reverse transcriptase inhibition by tiger milk mushroom (*Lignosus rhinocerus*) sclerotium extracts: In vitro and in silico studies. J. Tradit. Complementary Med..

[14] Esposito F., Carli I., Del Vecchio C., Xu L., Corona A., Grandi N., Piano D., Maccioni E., Distinto S., Parolin C. (2016). Sennoside A, derived from the traditional chinese medicine plant *Rheum* L., is a new dual HIV-1 inhibitor effective on HIV-1 replication. Phytomedicine.

[15] Zubair M.S., Maulana S., Widodo A., Mukaddas A., Pitopang R. (2020). Docking study on anti-HIV-1 activity of secondary metabolites from Zingiberaceae plants. J. Pharm. Bioallied Sci..

[16] Yuandani I.J., Rohani A.S., Sumantri I.B. (2021). Immunomodulatory effects and mechanisms of *Curcuma* species and their bioactive compounds: A review. Front. Pharmacol..

[17] Srivilai J., Khorana N., Waranuch N., Wisuitiprot W., Suphrom N., Suksamrarn A., Ingkaninan K. (2016). Germacrene analogs are anti-androgenic on androgen-dependent cells. Nat. Prod. Commun..

[18] Suphrom N., Pumthong G., Khorana N., Waranuch N., Limpeanchob N., Ingkaninan K. (2012). Anti-Androgenic effect of sesquiterpenes isolated from the rhizomes of *Curcuma aeruginosa* roxb. Fitoterapia.

[19] Hossain C.F., Al-Amin M., Sayem A.S.M., Siragee I.H., Tunan A.M., Hassan F., Kabir M.M., Sultana G.N.N. (2015). Antinociceptive principle from *Curcuma aeruginosa*. BMC Complementary Altern. Med..

[20] Kamazeri T.S.A.T., Abd Samah O., Taher M., Susanti D., Qaralleh H. (2012). Antimicrobial activity and essential oils of *Curcuma aeruginosa*, *Curcuma mangga*, and *Zingiber cassumunar* from Malaysia. Asian Pac. J. Trop. Med..

[21] Otake T., Mori H., Morimoto M., Ueba N., Sutardjo S., Kusumoto I.T., Hattori M., Namba T. (1995). Screening of Indonesian plant extracts for anti-human immunodeficiency virus—Type 1 (HIV-1) activity. Phytother. Res..

[22] Daussy C.F., Galais M., Pradel B., Robert-Hebmann V., Sagnier S., Pattingre S., Biard-Piechaczyk M., Espert L. (2020). HIV-1 Env induces pexophagy and an oxidative stress leading to uninfected CD4+ T cell death. Autophagy.

[23] Ezhilarasan D., Srilekha M., Raghu R. (2017). HAART and hepatotoxicity. J. Appl. Pharm. Sci..

[24] Carr A., Cooper D.A. (2000). Adverse effects of antiretroviral therapy. Lancet.

[25] Patridge E., Gareiss P., Kinch M.S., Hoyer D. (2016). An analysis of FDA-approved drugs: Natural products and their derivatives. Drug Discov. Today.

[26] Ahn K. (2017). The worldwide trend of using botanical drugs and strategies for developing global drugs. BMB Rep..

[27] Sookkongwaree K., Geitmann M., Roengsumran S., Petsom A., Danielson U.H. (2006). Inhibition of viral proteases by *Zingiberaceae* extracts and flavones isolated from *Kaempferia parviflora*. Die Pharm.-Int. J. Pharm. Sci..

[28] Yu Y.-B., Miyashiro H., Nakamura N., Hattori M., Park J.C. (2007). Effects of triterpenoids and flavonoids isolated from *Alnus firma* on HIV-1 viral enzymes. Arch. Pharmacal Res..

[29] Cheenpracha S., Karalai C., Ponglimanont C., Subhadhirasakul S., Tewtrakul S. (2006). Anti-HIV-1 protease activity of compounds from Boesenbergia pandurata. Bioorganic Med. Chem..

[30] Safe I.P., Amaral E.P., Araújo-Pereira M., Lacerda M.V., Printes V.S., Souza A.B., Beraldi-Magalhães F., Monteiro W.M., Sampaio V.S., Barreto-Duarte B. (2020). Adjunct N-acetylcysteine treatment in hospitalized patients with HIV-associated tuberculosis dampens the oxidative stress in peripheral blood: Results from the RIPENACTB Study trial. Front. Immunol..

[31] Moon-ai W., Niyomploy P., Boonsombat R., Sangvanich P., Karnchanatat A. (2012). A superoxide dismutase purified from the rhizome of *Curcuma aeruginosa* Roxb. as inhibitor of nitric oxide production in the macrophage-like RAW 264.7 cell line. Appl. Biochem. Biotechnol..

[32] Swain S., Rautray T.R. (2021). Estimation of trace elements, antioxidants, and antibacterial agents of regularly consumed Indian medicinal plants. Biol. Trace Elem. Res..

[33] Tonsomboon A., Prasanth M.I., Plaingam W., Tencomnao T. (2021). *Kaempferia parviflora* rhizome extract inhibits glutamate-induced toxicity in HT-22 mouse hippocampal neuronal cells and extends longevity in *Caenorhabditis elegans*. Biology.

[34] Simoh S., Zainal A. (2015). Chemical profiling of *Curcuma aeruginosa* Roxb. rhizome using different techniques of solvent extraction. Asian Pac. J. Trop. Biomed..

[35] Lipinski C.A., Lombardo F., Dominy B.W., Feeney P.J. (1997). Experimental and computational approaches to estimate solubility and permeability in drug discovery and development settings. Adv. Drug Deliv. Rev..

[36] Daina A., Michielin O., Zoete V. (2017). SwissADME: A free web tool to evaluate pharmacokinetics, drug-likeness and medicinal chemistry friendliness of small molecules. Sci. Rep..

[37] Rangsinth P., Sillapachaiyaporn C., Nilkhet S., Tencomnao T., Ung A.T., Chuchawankul S. (2021). Mushroom-Derived bioactive compounds potentially serve as the inhibitors of SARS-CoV-2 main protease: An in silico approach. J. Tradit. Complementary Med..

[38] Medina-O’Donnell M., Rivas F., Reyes-Zurita F.J., Cano-Muñoz M., Martinez A., Lupiañez J.A., Parra A. (2019). Oleanolic acid derivatives as potential inhibitors of HIV-1 protease. J. Nat. Prod..

[39] Xiao S., Tian Z., Wang Y., Si L., Zhang L., Zhou D. (2018). Recent progress in the antiviral activity and mechanism study of pentacyclic triterpenoids and their derivatives. Med. Res. Rev..

[40] Chang M.W., Ayeni C., Breuer S., Torbett B.E. (2010). Virtual screening for HIV protease inhibitors: A comparison of AutoDock 4 and Vina. PLoS ONE.

[41] Nguyen N.T., Nguyen T.H., Pham T.N.H., Huy N.T., Bay M.V., Pham M.Q., Nam P.C., Vu V.V., Ngo S.T. (2019). Autodock vina adopts more accurate binding poses but autodock4 forms better binding affinity. J. Chem. Inf. Modeling.

[42] Trott O., Olson A.J. (2010). AutoDock Vina: Improving the speed and accuracy of docking with a new scoring function, efficient optimization, and multithreading. J. Comput. Chem..

[43] Freis E.D., Stanton J.R., Litter J., Culbertson J.W., Halperin M.H., Moister F.C., Wilkins R.W. (1949). The hemodynamic effects of hypotensive drugs in man. II. Dihydroergocornine. J. Clin. Investig..

[44] Ravi S., Shanmugam B., Subbaiah G.V., Prasad S.H., Reddy K.S. (2017). Identification of food preservative, stress relief compounds by GC–MS and HR-LC/Q-TOF/MS; evaluation of antioxidant activity of *Acalypha indica* leaves methanolic extract (in vitro) and polyphenolic fraction (in vivo). J. Food Sci. Technol..

[45] Kumbhar S.T., Patil S.P., Une H.D. (2018). Phytochemical analysis of *Canna indica* L. roots and rhizomes extract. Biochem. Biophys. Rep..

[46] Pellicciari R., Passeri D., De Franco F., Mostarda S., Filipponi P., Colliva C., Gadaleta R.M., Franco P., Carotti A., Macchiarulo A. (2016). Discovery of 3α, 7α, 11β-trihydroxy-6α-ethyl-5β-cholan-24-oic acid (TC-100), a novel bile acid as potent and highly selective FXR agonist for enterohepatic disorders. J. Med. Chem..

[47] Pires D.E., Blundell T.L., Ascher D.B. (2015). pkCSM: Predicting small-molecule pharmacokinetic and toxicity properties using graph-based signatures. J. Med. Chem..

[48] Liu Z., Huang X., Hu L., Pham L., Poole K.M., Tang Y., Mahon B.P., Tang W., Li K., Goldfarb N.E. (2016). Effects of hinge-region natural polymorphisms on human immunodeficiency virus-type 1 protease structure, dynamics, and drug pressure evolution. J. Biol. Chem..

[49] Lansdon E.B., Liu Q., Leavitt S.A., Balakrishnan M., Perry J.K., Lancaster-Moyer C., Kutty N., Liu X., Squires N.H., Watkins W.J. (2011). Structural and binding analysis of pyrimidinol carboxylic acid and N-hydroxy quinazolinedione HIV-1 RNase H inhibitors. Antimicrob. Agents Chemother..

[50] Sillapachaiyaporn C., Rangsinth P., Nilkhet S., Ung A.T., Chuchawankul S., Tencomnao T. (2021). Neuroprotective effects against Glutamate-Induced HT-22 hippocampal cell damage and *Caenorhabditis elegans* lifespan/healthspan enhancing activity of *Auricularia polytricha* mushroom extracts. Pharmaceuticals.

[51] Panthong S., Boonsathorn N., Chuchawankul S. (2016). Antioxidant activity, anti-proliferative activity, and amino acid profiles of ethanolic extracts of edible mushrooms. Genet. Mol. Res..

