# Supplementary material for: HIV-1 Protease and Reverse Transcriptase Inhibitory Activities of Curcuma aeruginosa Roxb. Rhizome Extracts and the Phytochemical Profile Analysis: In Vitro and In Silico Screening
Source: Pharmaceuticals (Basel). 2021 Oct 31;14(11):1115. doi: 10.3390/ph14111115 (PMC8621417; doi:10.3390/ph14111115)
Supplement: Supplementary file 1 [file pharmaceuticals-14-01115-s001.zip › Supplementary data 1.pdf]

Supplementary data 1

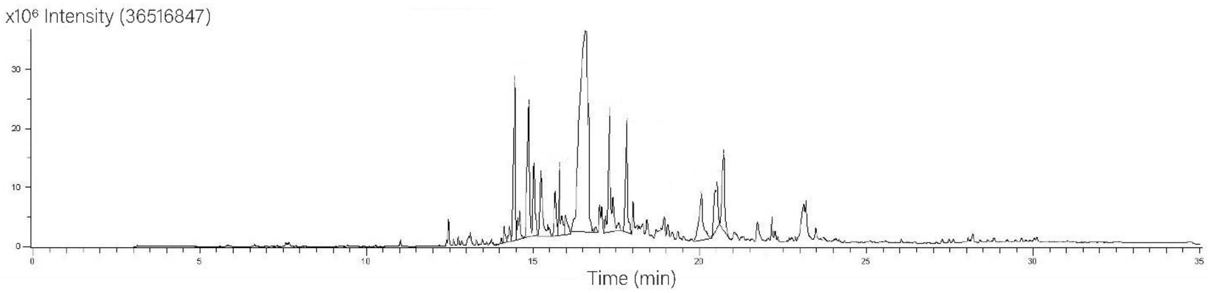

Figure S1 Chromatograms of chemical constituents presented in CA-H

Table S1 Phytochemical constituents in CA-H identified by GC-MS

Cpd #	RT	Mass	Name	Formula	Classification
Cpd 1	7.58	154	α-Terpineol	$C_{10}H_{18}O$	Terpenoids
Cpd 2	11.01	204	β-Elemene	$C_{15}H_{24}$	Terpenoids (Sesquiterpenoids)
Cpd 3	14.4	230	Cycloisolongifolene,8,9-dehydro-9-formyl-	$C_{16}H_{22}O$	Others
Cpd 4	14.85	220	9-Isopropyl-1-methyl-2-methylene-5-oxatricyclo[5.4.0.03,8]undecane	$C_{15}H_{24}O$	Others
Cpd 5	15.03	222	Cadinol T	$C_{15}H_{26}O$	Terpenoid (Terpene)
Cpd 6	15.24	222	α-Cadinol	$C_{15}H_{26}O$	Terpenoid (Terpene)
Cpd 7	15.66	232	4,7,7-Trimethyl-4-(2-methylallyl)tricyclo[3.3.0.02,8]octane-3,6-dione	$C_{15}H_{20}O_2$	Others
Cpd 8	16.52	234	3-(3,3,8,8-Tetramethyl-5-tricyclo[5.1.0.02,5]oct-5-enyl)propanoic acid	$C_{15}H_{22}O_2$	Others
Cpd 9	16.66	234	Dihydrocostunolide	$C_{15}H_{22}O_2$	Others
Cpd 10	17.0	234	4-(3,3-dimethylbut-1-ynyl)-4-hydroxy-2,6,6-trimethylcyclohex-2-en-1-one	$C_{15}H_{22}O_2$	Others
Cpd 11	17.29	206	(4Z)-4-(6,6-dimethyl-2-methylidenecyclohex-3-en-1-ylidene)pentan-2-ol	$C_{14}H_{22}O$	Others
Cpd 12	17.8	220	Isoaromadendrene epoxide	$C_{15}H_{24}O$	Others
Cpd 13	18.01	234	Cyclopropanebutanoic acid, 2-[[2-[[2-[(2-pentylcyclopropyl)methyl]cyclopropyl]methyl]	$C_{15}H_{22}O_2$	Others
Cpd 14	18.93	374	Palmitic acid	$C_{25}H_{42}O_2$	Fatty acid related compounds
Cpd 15	20.04	256	Xanthumin	$C_{16}H_{32}O_2$	Fatty acid related compounds

Cpd 16	20.7	306	6-(3-Hydroxyprop-1-en-2-yl)-4,8a-dimethyl-1,3,5,6,7,8-hexahydronaphthalen-2-one	$C_{17}H_{22}O_5$	Others
Cpd 17	20.45	234	Arglabin	$C_{15}H_{22}O_2$	Others
Cpd 18	20.52	234	Linoleic acid, methyl ester	$C_{15}H_{22}O_2$	Others
Cpd 19	21.75	246	Linoleic acid	$C_{15}H_{18}O_3$	Terpenoid (Terpene)
Cpd 20	22.17	294	Oleic Acid	$C_{19}H_{34}O_2$	Fatty acid related compound
Cpd 21	23.12	280	β-Levantenolide	$C_{18}H_{32}O_2$	Fatty acid related compound
Cpd 22	23.19	280	2,2,4-Trimethyl-3-[(3E,7E,11E)-3,8,12,16-tetramethylheptadeca-3,7,11,15-tetraenyl] cyclohexan-1-ol	$C_{18}H_{32}O_2$	Fatty acid related compound
Cpd 23	23.48	282	α-Terpineol	$C_{18}H_{34}O_2$	Fatty acid related compound
Cpd 24	28.05	318	β-Elemene	$C_{20}H_{30}O_3$	Others
Cpd 25	28.18	428	Cycloisolongifolene,8,9-dehydro-9-formyl-	$C_{30}H_{52}O$	Others
