# Supplementary material for: HIV-1 Protease and Reverse Transcriptase Inhibitory Activities of Curcuma aeruginosa Roxb. Rhizome Extracts and the Phytochemical Profile Analysis: In Vitro and In Silico Screening
Source: Pharmaceuticals (Basel). 2021 Oct 31;14(11):1115. doi: 10.3390/ph14111115 (PMC8621417; doi:10.3390/ph14111115)
Supplement: Supplementary file 1 [file pharmaceuticals-14-01115-s001.zip › Supplementary data 2.pdf]

Supplementary data 2

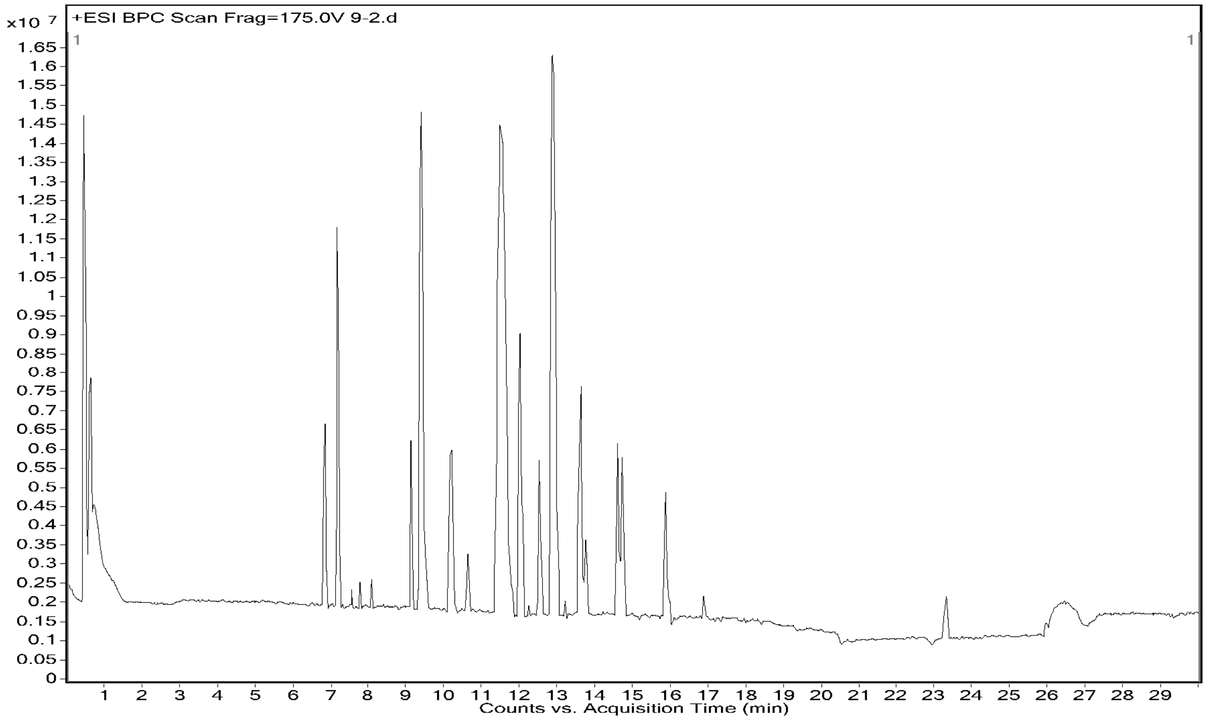

Figure S2 Chromatograms of chemical constituents presented in CA-EA

Table S2 Phytochemical constituents in CA-EA identified by UPLC-HRMS

Cpd #	RT	Mass	Name	Formula	Classification
Cpd 1	0.483	101.1218	N/A	-	-
Cpd 2	3.554	148.0733	Pantoic acid	$C_6H_{12}O_4$	Fatty acid related compound
Cpd 3	4.960	357.2585	Ile Leu Leu	$C_{18}H_{35}N_3O_4$	Amino acid related compound
Cpd 4	5.304	230.1877	13-Hydroxy-tridecanoic acid	$C_{13}H_{26}O_3$	Fatty acid related compound
Cpd 5	5.477	470.3407	(22S)-1α,25-Dihydroxy-22-methoxy-26,27-dimethyl-23,24-tetradehydro-20-epivitamin D3	$C_{30}H_{46}O_4$	Terpenoids (Sterol Lipids)
Cpd 6	6.356	226.1562	Dihydrojasmonic acid, methyl ester	$C_{13}H_{22}O_3$	Fatty acid related compound
Cpd 7	6.358	266.1485	Gemfibrozil M1	$C_{15}H_{22}O_4$	Others
Cpd 8	6.401	333.1680	Phe Ala Pro	$C_{17}H_{23}N_3O_4$	Amino acid related compounds
Cpd 9	6.514	217.2012	N/A	-	-
Cpd 10	6.807	261.2271	N/A	-	-
Cpd 11	6.829	244.1067	Pro Glu	$C_{10}H_{16}N_2O_5$	Amino acid related compound
Cpd 12	6.848	170.1305	Citronellic acid	$C_{10}H_{18}O_2$	Terpenoids

Cpd 13	6.849	182.1670	7E,9Z-Dodecadien-1-ol	$C_{12}H_{22}O$	Fatty acid related compound
Cpd 14	6.849	228.1718	3-oxo-Tridecanoic acid	$C_{13}H_{24}O_3$	Fatty acid related compound
Cpd 15	6.921	228.1716	10-keto Tridecanoic acid	$C_{13}H_{24}O_3$	Fatty acid related compound
Cpd 16	7.104	298.1380	Benzenehexanoic acid, 2,5dihydroxy-3,4-dimethoxy-6methyl-	$C_{15}H_{22}O_6$	Others
Cpd 17	7.112	297.1540	4-(2-Hydroxy-3isopropylaminoproxy) benzyloxy acetic acid	$C_{15}H_{23}NO_5$	Others
Cpd 18	7.199	248.1383	Amiloxate	$C_{15}H_{20}O_3$	Fatty acid related compound
Cpd 19	7.304	518.2278	Trp Gln Trp	$C_{27}H_{30}N_6O_5$	Amino acid related compound
Cpd 20	7.325	228.1721	3-oxo-Tridecanoic acid	$C_{13}H_{24}O_3$	Fatty acid related compound
Cpd 21	7.489	222.1252	Hydroxyibuprofen	$C_{13}H_{18}O_3$	Others
Cpd 22	7.570	226.1563	Dihydrojasmonic acid, methyl ester	$C_{13}H_{22}O_3$	Fatty acid related compound
Cpd 23	7.582	230.1876	13-Hydroxy-tridecanoic acid	$C_{13}H_{26}O_3$	Fatty acid related compound
Cpd 24	7.780	228.1720	3-oxo-Tridecanoic acid	$C_{13}H_{24}O_3$	Fatty acid related compound
Cpd 25	7.787	250.1537	Gemfibrozil	$C_{15}H_{22}O_3$	Fatty acid related compound
Cpd 26	7.817	246.1223	Val Glu	$C_{10}H_{18}N_2O_5$	Amino acid related compounds
Cpd 27	8.008	214.1566	2-oxo-Dodecanoic acid	$C_{12}H_{22}O_3$	Fatty acid related compound
Cpd 28	8.102	266.1486	Gemfibrozil M1	$C_{15}H_{22}O_4$	Others
Cpd 29	8.302	280.1278	Gemfibrozil M3	$C_{15}H_{20}O_5$	Fatty acid related compound
Cpd 30	8.559	245.2325	N/A	-	-
Cpd 31	8.844	230.1876	13-Hydroxy-tridecanoic acid	$C_{13}H_{26}O_3$	Fatty acid related compound
Cpd 32	8.998	228.1719	3-oxo-Tridecanoic acid	$C_{13}H_{24}O_3$	Fatty acid related compound
Cpd 33	9.148	200.1046	4Z-Decenedioic acid	$C_{10}H_{16}O_4$	Fatty acid related compound
Cpd 34	9.149	280.1278	Gemfibrozil M3	$C_{15}H_{20}O_5$	Fatty acid related compound
Cpd 35	9.155	228.1721	3-oxo-Tridecanoic acid	$C_{13}H_{24}O_3$	Fatty acid related compound
Cpd 36	9.408	212.1777	3-n-Decyl acrylic acid	$C_{13}H_{24}O_2$	Fatty acid related compound
Cpd 37	9.410	252.1696	Punctaporin B	$C_{15}H_{24}O_3$	Others
Cpd 38	9.466	246.1228	Val Glu	$C_{10}H_{18}N_2O_5$	Amino acid related compounds
Cpd 39	9.779	170.1307	Citronellic acid	$C_{10}H_{18}O_2$	Terpenoids
Cpd 40	9.813	230.1875	13-Hydroxy-tridecanoic acid	$C_{13}H_{26}O_3$	Amino acid related compounds
Cpd 41	10.069	157.1442	N/A	-	-
Cpd 42	10.170	273.2633	C16 Sphinganine	$C_{16}H_{35}NO_2$	Fatty acid related compound

Cpd 43	10.267	317.2888	Phytosphingosine	$C_{18}H_{39}NO_3$	Others
Cpd 44	10.587	248.1383	Amiloxate	$C_{15}H_{20}O_3$	Fatty acid related compound
Cpd 45	10.590	252.1695	Punctaporin B	$C_{15}H_{24}O_3$	Others
Cpd 46	10.659	338.3126	2,4-Dimethyl-2-eicosenoic acid	$C_{22}H_{42}O_2$	Fatty acid related compound
Cpd 47	10.661	228.1719	3-oxo-Tridecanoic acid	$C_{13}H_{24}O_3$	Fatty acid related compound
Cpd 48	10.887	252.1693	Punctaporin B	$C_{15}H_{24}O_3$	Others
Cpd 49	10.892	250.1535	Gemfibrozil	$C_{15}H_{22}O_3$	Fatty acid related compound
Cpd 50	11.286	270.1311	2-[3-Carboxy-3-(methylammonio)propyl]-L-histidine	$C_{11}H_{18}N_4O_4$	Others
Cpd 51	11.396	250.1536	Gemfibrozil	$C_{15}H_{22}O_3$	Fatty acid related compound
Cpd 52	11.397	188.1411	4-Hydroxy capric acid	$C_{10}H_{20}O_3$	Fatty acid related compound
Cpd 53	11.501	184.1829	9-Dodecen-1-ol	$C_{12}H_{24}O$	Fatty acid related compound (fatty alcohol)
Cpd 54	11.506	252.1699	Punctaporin B	$C_{15}H_{24}O_3$	Others
Cpd 55	11.512	128.1206	Octanal	$C_8H_{16}O$	Fatty acid related compound (fatty aldehyde)
Cpd 56	11.522	170.1308	Citronellic acid	$C_{10}H_{18}O_2$	Terpenoids
Cpd 57	11.532	170.1671	Undecanal	$C_{11}H_{22}O$	Fatty acid related compound (fatty aldehyde)
Cpd 58	11.532	156.1155	6E-Nonenoic acid	$C_9H_{16}O_2$	Fatty acid related compound
Cpd 59	11.532	114.1052	4-Heptanone	$C_7H_{14}O$	Fatty acid related compound (ketone)
Cpd 60	11.534	100.0895	4-Methylpentanal	$C_6H_{12}O$	Others (Carbohydrate)
Cpd 61	11.538	230.1878	13-Hydroxy-tridecanoic acid	$C_{13}H_{26}O_3$	Fatty acid related compound
Cpd 62	11.542	212.1776	3-n-Decyl acrylic acid	$C_{13}H_{24}O_2$	Fatty acid related compound
Cpd 63	11.609	301.2946	Dihydrosphingosine	$C_{18}H_{39}NO_2$	Fatty acid related compound
Cpd 64	11.797	356.1225	Deoxysappanone B 7,3'- dimethyl ether acetate	$C_{20}H_{20}O_6$	Others
Cpd 65	11.807	320.1953	QH2	$C_{19}H_{28}O_4$	Others
Cpd 66	12.025	232.1437	Ile Thr	$C_{10}H_{20}N_2O_4$	Amino acid related compound
Cpd 67	12.032	224.1409	Methyl jasmonate	$C_{13}H_{20}O_3$	Terpenoids
Cpd 68	12.032	246.1225	Val Glu	$C_{10}H_{18}N_2O_5$	Amino acid related compounds
Cpd 69	12.032	134.0584	Deoxyribose	$C_5H_{10}O_4$	Others (Carbohydrate)
Cpd 70	12.113	212.1774	3-n-Decyl acrylic acid	$C_{13}H_{24}O_2$	Fatty acid related compound
Cpd 71	12.277	212.1775	3-n-Decyl acrylic acid	$C_{13}H_{24}O_2$	Fatty acid related compound
Cpd 72	12.552	470.1650	7-Hydroxymethotrexate	$C_{20}H_{22}N_8O_6$	Others

Cpd 73	12.552	114.1051	4-Heptanone	$C_7H_{14}O$	Fatty acid related compound (ketone)
Cpd 74	12.553	432.2100	6β,11β,16α,17α,21-Pentahydroxypregna-1,4-diene-3,20-dione-16,17-acetonide	$C_{24}H_{32}O_7$	Terpenoids
Cpd 75	12.553	449.2364	Phe Gln Arg	$C_{20}H_{31}N_7O_5$	Amino acid related compound
Cpd 76	12.896	212.1775	3-n-Decyl acrylic acid	$C_{13}H_{24}O_2$	Fatty acid related compound
Cpd 77	12.954	246.1230	Ile Asp	$C_{10}H_{18}N_2O_5$	Amino acid related compound
Cpd 78	13.011	329.3258	N/A	-	-
Cpd 79	13.233	212.1775	3-n-Decyl acrylic acid	$C_{13}H_{24}O_2$	Fatty acid related compound
Cpd 80	13.270	230.1883	13-Hydroxy-tridecanoic acid	$C_{13}H_{26}O_3$	Fatty acid related compound
Cpd 81	13.579	212.1775	3-n-Decyl acrylic acid	$C_{13}H_{24}O_2$	Fatty acid related compound
Cpd 82	13.643	144.0789	Hydroxycyclohexanecarboxylic acid	$C_7H_{12}O_3$	Others
Cpd 83	13.644	226.1565	Dihydrojasmonic acid, methyl ester	$C_{13}H_{22}O_3$	Fatty acid related compound
Cpd 84	13.760	212.1773	3-n-Decyl acrylic acid	$C_{13}H_{24}O_2$	Fatty acid related compound
Cpd 85	13.762	252.1697	Punctaporin B	$C_{15}H_{24}O_3$	Others
Cpd 86	13.939	226.1566	Dihydrojasmonic acid, methyl ester	$C_{13}H_{22}O_3$	Fatty acid related compound
Cpd 87	14.174	212.1774	3-n-Decyl acrylic acid	$C_{13}H_{24}O_2$	Fatty acid related compound
Cpd 88	14.175	226.1566	Dihydrojasmonic acid, methyl ester	$C_{13}H_{22}O_3$	Fatty acid related compound
Cpd 89	14.513	354.2362	Prostaglandin H1	$C_{20}H_{34}O_5$	Fatty acid related compound
Cpd 90	15.890	296.1591	Lactone of PGF-MUM	$C_{16}H_{24}O_5$	Fatty acid related compound
Cpd 91	15.892	144.0427	(E)-2-Methylglutaconic acid	$C_6H_8O_4$	Fatty acid related compound
Cpd 92	16.900	296.2340	12-Hydroxy-10-octadecynoic acid	$C_{18}H_{32}O_3$	Fatty acid related compound
Cpd 93	18.430	397.2208	GPEtn(12:0/0:0)	$C_{17}H_{36}NO_7P$	Fatty acid related compound
Cpd 94	18.865	438.3291	27-nor-5β-Cholestane-3α,7α,12α,24,25-pentol	$C_{26}H_{46}O_5$	Terpenoid (Sterol Lipids)
Cpd 95	19.369	430.2650	Gln Lys Arg	$C_{17}H_{34}N_8O_5$	Amino acid related compound
Cpd 96	23.316	430.2651	Gln Lys Arg	$C_{17}H_{34}N_8O_5$	Amino acid related compound
Cpd 97	23.317	408.2831	3β,6α,7α -Trihydroxy-5β-cholan-24-oic acid	$C_{24}H_{40}O_5$	Terpenoid (Sterol Lipids)
Cpd 98	25.986	141.9921	N/A	-	-
Cpd 99	26.000	125.0135	Taurine	$C_2H_7NO_3S$	Amino acid related compound
Cpd 100	26.842	125.9983	2-Hydroxyethanesulfonate	$C_2H_6O_4S$	Amino acid related compound

N/A indicates not applicable of non-identified compound
