# Supplementary material for: HIV-1 Protease and Reverse Transcriptase Inhibitory Activities of Curcuma aeruginosa Roxb. Rhizome Extracts and the Phytochemical Profile Analysis: In Vitro and In Silico Screening
Source: Pharmaceuticals (Basel). 2021 Oct 31;14(11):1115. doi: 10.3390/ph14111115 (PMC8621417; doi:10.3390/ph14111115)
Supplement: Supplementary file 1 [file pharmaceuticals-14-01115-s001.zip › Supplementary data 3.pdf]

**Supplementary data 3**

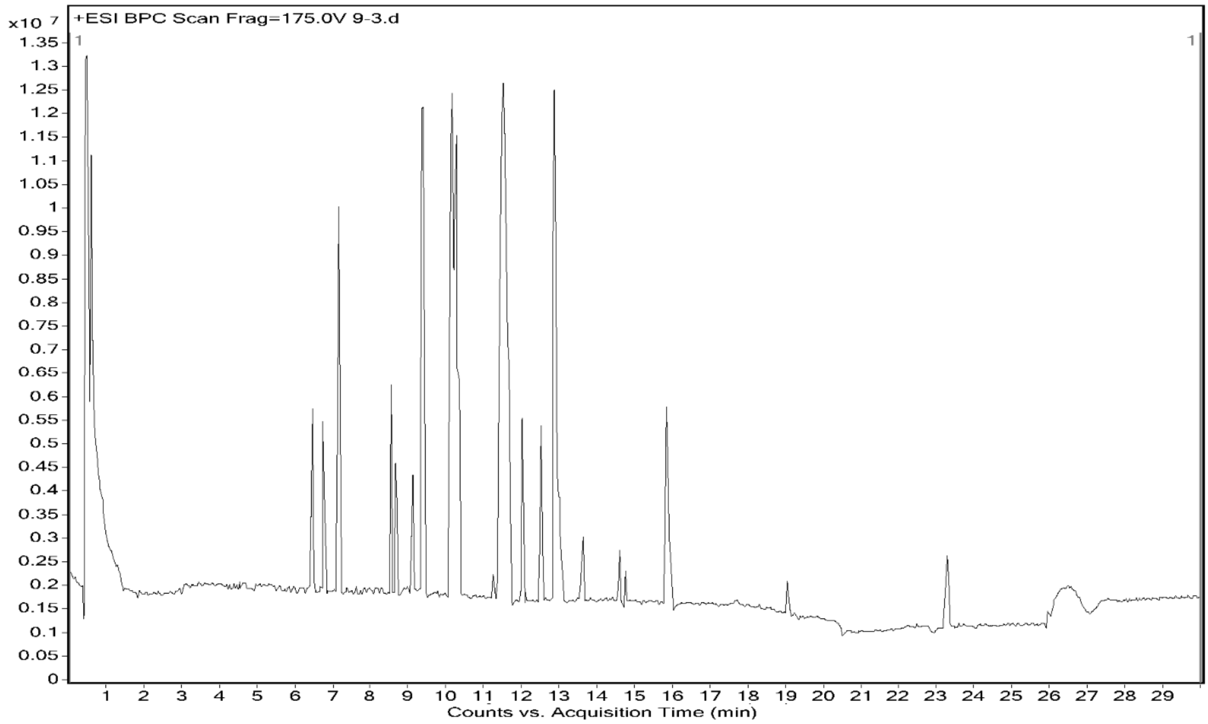


**Figure S3** Chromatograms of chemical constituents presented in CA-M

**Table S3** Phytochemical constituents in CA-M identified by UPLC-HRMS

| **Cpd #** | **RT** | **Mass** | **Name** | **Formula** | **Classification** |
|---|---|---|---|---|---|
| Cpd 1 | 0.472 | 360.1225 | Elephantopin | $C_{19}H_{20}O_7$ | Terpenoids |
| Cpd 2 | 0.489 | 238.2148 | N/A | - | - |
| Cpd 3 | 0.539 | 118.0851 | Betaine | $C_5H_{12}NO_2$ | Amino acid related compounds |
| Cpd 4 | 1.867 | 131.0931 | Leucine | $C_6H_{13}NO_2$ | Amino acid related compounds |
| Cpd 5 | 2.920 | 188.0663 | Ethyl oxalacetate | $C_8H_{12}O_5$ | Fatty acids related compounds |
| Cpd 6 | 4.909 | 357.2589 | Ile Leu Leu | $C_{18}H_{35}N_3O_4$ | Amino acid related compounds |
| Cpd 7 | 5.829 | 559.3736 | (6R)-Vitamin D3 6,19-(4-phenyl-1,2,4-triazoline-3,5-dione) adduct / (6R)-cholecalciferol 6,19-(4-phenyl-1,2,4-triazoline-3,5-dione) adduct | $C_{35}H_{49}N_3O_3$ | Terpenoids |
| Cpd 8 | 6.034 | 607.3711 | N/A | - | - |
| Cpd 9 | 6.346 | 355.1503 | Ala Glu His | $C_{14}H_{21}N_5O_6$ | Amino acid related compounds |
| Cpd 10 | 6.469 | 217.2016 | N/A | | |
| Cpd 11 | 6.777 | 261.2276 | N/A | | |

| | | | | | |
|---|---|---|---|---|---|
| Cpd 12 | 6.808 | 170.131 | Citronellic acid | $C_{10}H_{18}O_2$ | Terpenoids |
| Cpd 13 | 6.814 | 228.1722 | 3-oxo-Tridecanoic acid | $C_{13}H_{24}O_3$ | Fatty acids related compounds |
| Cpd 14 | 6.836 | 226.1568 | Dihydrojasmonic acid, methyl ester | $C_{13}H_{22}O_3$ | Fatty acids related compounds |
| Cpd 15 | 6.870 | 250.1541 | Gemfibrozil | $C_{15}H_{22}O_3$ | Fatty acids related compounds |
| Cpd 16 | 7.025 | 305.2531 | N/A | - | - |
| Cpd 17 | 7.052 | 298.1387 | Benzenehexanoic acid, 2,5-dihydroxy-3,4-dimethoxy-6-methyl- | $C_{15}H_{22}O_6$ | Others |
| Cpd 18 | 7.167 | 226.1567 | Dihydrojasmonic acid, methyl ester | $C_{13}H_{22}O_3$ | Fatty acids related compounds |
| Cpd 19 | 7.297 | 228.1722 | 3-oxo-Tridecanoic acid | $C_{13}H_{24}O_3$ | Fatty acids related compounds |
| Cpd 20 | 7.453 | 222.1254 | Hydroxyibuprofen | $C_{13}H_{18}O_3$ | Others |
| Cpd 21 | 7.547 | 226.1567 | Dihydrojasmonic acid, methyl ester | $C_{13}H_{22}O_3$ | Fatty acids related compounds |
| Cpd 22 | 7.560 | 230.1879 | 13-Hydroxy-tridecanoic acid | $C_{13}H_{26}O_3$ | Fatty acids related compounds |
| Cpd 23 | 7.755 | 250.1541 | Gemfibrozil | $C_{15}H_{22}O_3$ | Fatty acids related compounds |
| Cpd 24 | 7.793 | 224.1409 | Methyl jasmonate | $C_{13}H_{20}O_3$ | Terpenoids |
| Cpd 25 | 8.069 | 266.149 | Gemfibrozil M1 | $C_{15}H_{22}O_4$ | Others |
| Cpd 26 | 8.087 | 228.1722 | 3-oxo-Tridecanoic acid | $C_{13}H_{24}O_3$ | Fatty acids related compounds |
| Cpd 27 | 8.553 | 245.2328 | N/A | - | - |
| Cpd 28 | 8.831 | 230.1881 | 13-Hydroxy-tridecanoic acid | $C_{13}H_{26}O_3$ | Fatty acids related compounds |
| Cpd 29 | 8.843 | 333.284 | N/A | | |
| Cpd 30 | 9.122 | 280.1282 | Gemfibrozil M3 | $C_{15}H_{20}O_5$ | Fatty acids related compounds |
| Cpd 31 | 9.394 | 184.1831 | 9-Dodecen-1-ol | $C_{12}H_{24}O$ | Fatty acid related compound (fatty alcohol) |
| Cpd 32 | 9.395 | 212.1775 | 3-n-Decyl acrylic acid | $C_{13}H_{24}O_2$ | Fatty acid related compound |
| Cpd 33 | 9.396 | 252.17 | Punctaporin B | $C_{15}H_{24}O_3$ | Others |
| Cpd 34 | 9.525 | 563.3116 | Dihydroergocornine | $C_{31}H_{41}N_5O_5$ | Others |
| Cpd 35 | 9.526 | 568.2669 | Swietenine | $C_{32}H_{40}O_9$ | Terpenoids |
| Cpd 36 | 9.781 | 230.188 | 13-Hydroxy-tridecanoic acid | $C_{13}H_{26}O_3$ | Fatty acids related compounds |
| Cpd 37 | 9.840 | 450.2204 | Methyldopexamine sulfate | $C_{23}H_{34}N_2O_5S$ | Others |
| Cpd 38 | 9.852 | 248.1386 | Amiloxate | $C_{15}H_{20}O_3$ | Fatty acids related compounds |
| Cpd 39 | 10.045 | 157.1446 | N/A | - | - |
| Cpd 40 | 10.163 | 273.2639 | Hexadecasphinganine | $C_{16}H_{35}NO_2$ | Fatty acid related compound |
| Cpd 41 | 10.212 | 210.1623 | 3-Tridecynoic acid | $C_{13}H_{22}O_2$ | Fatty acids related compounds |
| Cpd 42 | 10.221 | 229.2382 | N/A | - | - |
| Cpd 43 | 10.271 | 317.2896 | Phytosphingosine | $C_{18}H_{39}NO_3$ | Fatty acid related compound |
| Cpd 44 | 10.359 | 357.3438 | N-(2-fluro-ethyl)-eicosanoyl amine | $C_{22}H_{44}FNO$ | Fatty acid related compound |

| | | | | | |
|---|---|---|---|---|---|
| Cpd 45 | 10.361 | 333.2843 | N/A | - | - |
| Cpd 46 | 10.361 | 289.2586 | N/A | - | - |
| Cpd 47 | 10.650 | 342.2845 | Prostaglandin F1a alcohol | $C_{20}H_{38}O_4$ | Fatty acids related compounds |
| Cpd 48 | 10.658 | 228.1724 | 3-oxo-Tridecanoic acid | $C_{13}H_{24}O_3$ | Fatty acids related compounds |
| Cpd 49 | 10.885 | 250.1546 | Gemfibrozil | $C_{15}H_{22}O_3$ | Fatty acids related compounds |
| Cpd 50 | 11.084 | 196.1467 | 3-Dodecynoic acid | $C_{12}H_{20}O_2$ | Fatty acids related compounds |
| Cpd 51 | 11.108 | 144.0428 | (E)-2-Methylglutaconic acid | $C_6H_8O_4$ | Fatty acids related compounds |
| Cpd 52 | 11.271 | 270.1316 | 2-[3-Carboxy-3-(methylammonio)propyl]-L-histidine | $C_{11}H_{18}N_4O_4$ | Amino acid related compound |
| Cpd 53 | 11.496 | 184.183 | 9-Dodecen-1-ol | $C_{12}H_{24}O$ | Fatty acid related compound (fatty alcohol) |
| Cpd 54 | 11.502 | 252.1701 | Punctaporin B | $C_{15}H_{24}O_3$ | Others |
| Cpd 55 | 11.509 | 128.1209 | Octanal | $C_8H_{16}O$ | Fatty acid related compound (fatty aldehyde) |
| Cpd 56 | 11.511 | 170.1311 | Citronellic acid | $C_{10}H_{18}O_2$ | Terpenoids |
| Cpd 57 | 11.516 | 156.1157 | 6E-Nonenoic acid | $C_9H_{16}O_2$ | Fatty acids related compounds |
| Cpd 58 | 11.518 | 100.0898 | 4-Methylpentanal | $C_6H_{12}O$ | Others (Carbohydrate) |
| Cpd 59 | 11.520 | 230.1881 | 13-Hydroxy-tridecanoic acid | $C_{13}H_{26}O_3$ | Fatty acids related compounds |
| Cpd 60 | 11.522 | 212.1777 | 3-n-Decyl acrylic acid | $C_{13}H_{24}O_2$ | Fatty acids related compounds |
| Cpd 61 | 11.613 | 301.2949 | Dihydrosphingosine | $C_{18}H_{39}NO_2$ | Fatty acid related compound |
| Cpd 62 | 11.689 | 345.3204 | N/A | - | - |
| Cpd 63 | 11.759 | 389.3462 | N/A | - | - |
| Cpd 64 | 11.807 | 320.1955 | QH2 | $C_{19}H_{28}O_4$ | Others |
| Cpd 65 | 12.040 | 210.162 | 3-Tridecynoic acid | $C_{13}H_{22}O_2$ | Fatty acids related compounds |
| Cpd 66 | 12.045 | 246.1229 | Val Glu | $C_{10}H_{18}N_2O_5$ | Amino acid related compounds |
| Cpd 67 | 12.046 | 134.0587 | Deoxyribose | $C_5H_{10}O_4$ | Others (Carbohydrate) |
| Cpd 68 | 12.046 | 224.1413 | Methyl jasmonate | $C_{13}H_{20}O_3$ | Terpenoids |
| Cpd 69 | 12.115 | 212.1778 | 3-n-Decyl acrylic acid | $C_{13}H_{24}O_2$ | Fatty acids related compounds |
| Cpd 70 | 12.539 | 432.2102 | 6β,11β,16α,17α,21-Pentahydroxypregna-1,4-diene-3,20-dione-16,17-acetonide | $C_{24}H_{32}O_7$ | Terpenoids |
| Cpd 71 | 12.542 | 392.218 | Phe Ala Arg | $C_{18}H_{28}N_6O_4$ | Amino acid related compounds |
| Cpd 72 | 12.542 | 449.2368 | Phe Gln Arg | $C_{20}H_{31}N_7O_5$ | Amino acid related compounds |
| Cpd 73 | 12.542 | 114.1053 | 4-Heptanone | $C_7H_{14}O$ | Fatty acid related compound (ketone) |
| Cpd 74 | 12.542 | 470.1657 | 7-Hydroxymethotrexate | $C_{20}H_{22}N_8O_6$ | Others |
| Cpd 75 | 12.894 | 216.1491 | Val Val | $C_{10}H_{20}N_2O_3$ | Amino acid related compounds |

| | | | | | |
|---|---|---|---|---|---|
| Cpd 76 | 13.008 | 329.3258 | N/A | - | - |
| Cpd 77 | 13.068 | 373.3518 | N/A | - | - |
| Cpd 78 | 13.138 | 417.3773 | N/A | - | - |
| Cpd 79 | 13.221 | 212.1778 | 3-n-Decyl acrylic acid | $C_{13}H_{24}O_2$ | Fatty acids related compounds |
| Cpd 80 | 13.372 | 355.341 | N-(2-Hydroxyethyl) icosanamide | $C_{22}H_{45}NO_2$ | Fatty acids related compounds |
| Cpd 81 | 13.419 | 399.3667 | N/A | - | - |
| Cpd 82 | 13.639 | 226.157 | Dihydrojasmonic acid, methyl ester | $C_{13}H_{22}O_3$ | Fatty acids related compounds |
| Cpd 83 | 13.639 | 144.0794 | Hydroxycyclohexanecarboxylic acid | $C_7H_{12}O_3$ | Others |
| Cpd 84 | 13.757 | 252.17 | Punctaporin B | $C_{15}H_{24}O_3$ | Others |
| Cpd 85 | 13.940 | 226.1571 | Dihydrojasmonic acid, methyl ester | $C_{13}H_{22}O_3$ | Fatty acids related compounds |
| Cpd 86 | 14.518 | 354.2371 | Prostaglandin H1 | $C_{20}H_{34}O_5$ | Fatty acids related compounds |
| Cpd 87 | 14.652 | 212.1778 | 3-n-Decyl acrylic acid | $C_{13}H_{24}O_2$ | Fatty acids related compounds |
| Cpd 88 | 15.448 | 384.247 | Ibutilide | $C_{20}H_{36}N_2O_3S$ | Others |
| Cpd 89 | 15.876 | 296.1591 | Lactone of PGF-MUM | $C_{16}H_{24}O_5$ | Terpenoids |
| Cpd 90 | 15.877 | 144.0427 | (E)-2-Methylglutaconic acid | $C_6H_8O_4$ | Fatty acids related compounds |
| Cpd 91 | 16.902 | 296.2343 | 12-Hydroxy-10-octadecynoic acid | $C_{18}H_{32}O_3$ | Fatty acids related compounds |
| Cpd 92 | 18.112 | 424.3141 | N/A | - | - |
| Cpd 93 | 18.422 | 397.2211 | GPEtn(12:0/0:0) | $C_{17}H_{36}NO_7P$ | Fatty acid related compound |
| Cpd 94 | 18.966 | 482.3551 | N/A | - | - |
| Cpd 95 | 19.027 | 438.3294 | 27-nor-5β-Cholestane-3α,7α,12α,24,25-pentol | $C_{26}H_{46}O_5$ | Terpenoid (Sterol Lipids) |
| Cpd 96 | 19.094 | 434.2963 | 1α-Hydroxy-24-(dimethylphosphoryl)-25,26,27-trinorvitamin D3 | $C_{26}H_{43}O_3P$ | Terpenoids |
| Cpd 97 | 23.284 | 430.2653 | Gln Lys Arg | $C_{17}H_{34}N_8O_5$ | Amino acid related compounds |
| Cpd 98 | 23.287 | 408.2832 | 3β,6α,7α -Trihydroxy-5β-cholan-24-oic acid | $C_{24}H_{40}O_5$ | Terpenoids (steroid) |
| Cpd 99 | 26.000 | 141.9924 | N/A | - | - |
| Cpd 100 | 26.017 | 125.0135 | Taurine | $C_2H_7NO_3S$ | Amino acid related compounds |

N/A indicates not applicable of non-identified compound
