# Supplementary material for: HIV-1 Protease and Reverse Transcriptase Inhibitory Activities of Curcuma aeruginosa Roxb. Rhizome Extracts and the Phytochemical Profile Analysis: In Vitro and In Silico Screening
Source: Pharmaceuticals (Basel). 2021 Oct 31;14(11):1115. doi: 10.3390/ph14111115 (PMC8621417; doi:10.3390/ph14111115)
Supplement: Supplementary file 1 [file pharmaceuticals-14-01115-s001.zip › Supplementary data 4.pdf]

Supplementary data 4

Table S4 Prediction of Lipinski's rule of five, GI absorption, and toxicity parameters of identified compounds from CA extracts

Name	MW	#Rotatable bonds	#H-bond acceptors	#H-bond donors	MLOGP	Lipinski #violation	GI absorption	AMES test	Hepatotoxicity
(22S)-1α,25-Dihydroxy-22-methoxy-26,27-dimethyl-23,24-tetradehydro-20-epivitamin D3	470.68	6	4	3	3.97	0	High	No	Yes
(4Z)-4-(6,6-Dimethyl-2-methylidenecyclohex-3-en-1-ylidene)pentan-2-ol	206.32	2	1	1	3.21	0	High	No	No
(6R)-Vitamin D3 6,19-(4-phenyl-1,2,4-triazoline-3,5-dione) adduct / (6R)-cholecalciferol 6,19-(4-phenyl-1,2,4-triazoline-3,5-dione) adduct	559.78	7	3	1	6.33	2	Low	No	Yes
(E)-2-Methylglutaconic acid	144.13	3	4	2	0.15	0	High	No	No
10-keto Tridecanoic acid	228.33	11	3	1	2.44	0	High	No	No
12-Hydroxy-10-octadecynoic acid	296.44	13	3	2	3.69	0	High	No	No
13-Hydroxy-tridecanoic acid	230.34	12	3	2	2.54	0	High	No	No
1α-Hydroxy-24-(dimethylphosphoryl)-25,26,27-trinorvitamin D3	434.59	6	3	2	4.03	0	High	No	Yes
2,2,4-Trimethyl-3-[(3E,7E,11E)-3,8,12,16-tetramethylheptadeca-3,7,11,15-tetraenyl] cyclohexan-1-ol	428.73	12	1	1	6.63	1	Low	No	No
2,4-Dimethyl-2-eicosenoic acid	338.57	17	2	1	5.47	1	Low	No	Yes
2-[3-Carboxy-3-(methylammonio)propyl]-L-histidine	270.29	8	7	5	-6.03	0	Low	No	Yes
27-nor-5β-Cholestane-3α,7α,12α,24,25-pentol	438.64	5	5	5	2.79	0	High	No	No
2-Hydroxyethanesulfonate	126.13	2	4	2	-1.51	0	High	No	No
2-oxo-Dodecanoic acid	214.3	10	3	1	2.16	0	High	No	No
3-(3,3,8,8-Tetramethyl-5-tricyclo[5.1.0.02,5]oct-5-enyl)propanoic acid	234.33	3	2	1	3.44	0	High	No	No
3-Dodecynoic acid	196.29	7	2	1	3.04	0	High	No	No
3-n-Decyl acrylic acid	212.33	10	2	1	3.32	0	High	No	No
3-oxo-Tridecanoic acid	228.33	11	3	1	2.44	0	High	No	No
3-Tridecynoic acid	210.31	8	2	1	3.32	0	High	No	No
3β,6α,7α -Trihydroxy-5β-cholan-24-oic acid	408.57	4	5	4	3.05	0	High	No	No

Name	MW	#Rotatable bonds	#H-bond acceptors	#H-bond donors	MLOGP	Lipinski #violation	GI absorption	AMES test	Hepatotoxicity
4-(2-Hydroxy-3isopropylaminoproxy) benzyloxy acetic acid	297.35	10	6	3	0.54	0	High	No	Yes
4-(3,3-dimethylbut-1-ynyl)-4-hydroxy-2,6,6-trimethylcyclohex-2-en-1-one	234.33	0	2	1	2.54	0	High	No	No
4,7,7-Trimethyl-4-(2-methylallyl)tricyclo[3.3.0.02,8]octane-3,6-dione	232.32	2	2	0	2.54	0	High	No	No
4-Heptanone	114.19	4	1	0	1.74	0	High	No	No
4-Hydroxy capric acid	188.26	8	3	2	1.7	0	High	No	No
4-Methylpentanal	100.16	3	1	0	1.39	0	High	No	No
4Z-Decenedioic acid	200.23	8	4	2	1.46	0	High	No	No
6-(3-Hydroxyprop-1-en-2-yl)-4,8a-dimethyl-1,3,5,6,7,8-hexahydronaphthalen-2-one	234.33	2	2	1	2.54	0	High	No	No
6E-Nonenoic acid	156.22	6	2	1	2.17	0	High	No	Yes
6β,11β,16α,17α,21-Pentahydroxypregna-1,4-diene-3,20-dione-16,17-acetonide	432.51	2	7	3	0.74	0	High	No	No
7E,9Z-Dodecadien-1-ol	182.3	8	1	1	3.16	0	High	No	No
7-Hydroxymethotrexate	470.44	10	9	6	-1.54	2	Low	No	Yes
9-Dodecen-1-ol	184.32	9	1	1	3.27	0	High	No	No
9-Isopropyl-1-methyl-2-methylene-5-oxatricyclo[5.4.0.03,8]undecane	220.35	1	1	0	3.67	0	High	No	No
Ala Glu His	355.35	12	8	6	-2.45	2	Low	No	Yes
Amiloxate	248.32	7	3	0	2.95	0	High	No	No
Arglabin	246.3	0	3	0	2.47	0	High	No	No
Benzenehexanoic acid, 2,5-dihydroxy-3,4-dimethoxy-6-methyl-	298.33	8	6	3	1.35	0	High	No	No
Betaine	117.15	2	2	0	-3.67	0	Low	No	No
Cadinol T	222.37	1	1	1	3.67	0	High	No	No
Citronellic acid	170.25	5	2	1	2.47	0	High	No	No
Cycloisolongifolene,8,9-dehydro-9-formyl-	230.35	1	1	0	3.81	0	High	No	No
Cyclopropanebutanoic acid, 2-[[2-[[2-[(2-pentylcyclopropyl)methyl]cyclopropyl]methyl]	374.6	15	2	0	5.82	1	Low	No	No
Deoxyribose	134.13	4	4	3	-1.65	0	High	No	No
Deoxysappanone B 7,3'- dimethyl ether acetate	356.37	6	6	0	1.82	0	High	Yes	No

Name	MW	#Rotatable bonds	#H-bond acceptors	#H-bond donors	MLOGP	Lipinski #violation	GI absorption	AMES test	Hepatotoxicity
Dihydrocostunolide	234.33	0	2	0	3.35	0	High	No	No
Dihydroergocornine	563.69	5	6	3	0.95	1	High	No	No
Dihydrojasmonic acid, methyl ester	226.31	7	3	0	2.04	0	High	No	No
Dihydrosphingosine	301.51	16	3	3	3.13	0	High	No	No
Elephantopin	360.36	3	7	0	1.5	0	High	Yes	No
Ethyl Oxalacetate	188.18	7	5	0	0.01	0	High	No	No
Gemfibrozil	250.33	6	3	1	3.04	0	High	No	No
Gemfibrozil M1	266.33	7	4	2	2.19	0	High	No	No
Gemfibrozil M3	280.32	7	5	2	2.35	0	High	No	No
Gln Lys Arg	430.5	18	8	8	-2.28	2	Low	No	No
GPEtn(12:0/0:0)	397.44	19	8	3	0.99	0	Low	No	No
Hexadecasphinganine	273.45	14	3	3	2.65	0	High	No	No
Hydroxycyclohexanecarboxylic acid	144.17	1	3	2	0.35	0	High	No	Yes
Hydroxyibuprofen	222.28	4	3	2	2.25	0	High	No	No
Ibutilide	384.58	14	4	2	2.46	0	High	No	Yes
Ile Asp	246.26	8	6	4	-0.58	0	High	Yes	No
Ile Leu Leu	357.49	13	5	4	1.11	0	High	No	No
Ile Thr	232.28	7	5	4	-0.49	0	High	No	No
Isoaromadendrene epoxide	220.35	0	1	0	3.81	0	High	No	No
Lactone of PGF-MUM	296.36	8	5	1	1.85	0	High	No	No
Leucine	131.17	3	3	2	-1.82	0	High	No	No
Linoleic acid	280.45	14	2	1	4.47	1	High	No	Yes
Linoleic acid, methyl ester	294.47	15	2	0	4.7	1	High	No	No
Methyl jasmonate	224.3	6	3	0	1.95	0	High	No	No
Methyldopexamine sulfate	450.59	16	7	3	2.75	0	High	No	Yes
N-(2-fluro-ethyl)-eicosanoyl amine	357.59	21	2	1	5.28	1	Low	No	No
N-(2-hydroxyethyl) icosanamide	355.6	21	2	2	4.29	1	High	No	No
Octanal	128.21	6	1	0	2.07	0	High	No	NO
Oleic Acid	282.46	15	2	1	4.57	1	High	No	No

Name	MW	#Rotatable bonds	#H-bond acceptors	#H-bond donors	MLOGP	Lipinski #violation	GI absorption	AMES test	Hepatotoxicity
Palmitic acid	256.42	14	2	1	4.19	1	High	No	No
Pantoic acid	148.16	3	4	3	-0.46	0	High	No	No
Phe Ala Arg	392.45	13	6	6	-0.35	1	Low	No	Yes
Phe Ala Pro	333.38	8	5	3	0.22	0	High	No	Yes
Phe Gln Arg	449.5	16	7	7	-1.08	2	Low	No	Yes
Phytosphingosine	317.51	16	4	4	2.28	0	High	No	No
Pro Glu	244.24	7	6	4	-0.97	0	High	No	No
Prostaglandin F1a alcohol	342.51	13	4	4	2.2	0	High	No	No
Prostaglandin H1	354.48	13	5	2	2.89	0	High	No	No
Punctaporin B	252.35	1	3	3	1.75	0	High	No	No
QH2	320.42	7	4	2	3.06	0	High	No	No
Swietenine	568.65	7	9	1	2.41	1	Low	No	Yes
Taurine	125.15	2	4	2	-1.51	0	High	No	No
Trp Gln Trp	518.56	14	6	7	-0.35	3	Low	No	Yes
Undecanal	170.29	9	1	0	2.99	0	High	No	No
Val Glu	246.26	8	6	4	-0.58	0	High	Yes	No
Val Val	216.28	6	4	3	0.32	0	High	No	No
Xanthumin	306.35	5	5	0	1.92	0	High	No	No
α-Cadinol	222.37	1	1	1	3.67	0	High	No	No
α-Terpineol	154.25	1	1	1	2.3	0	High	No	No
β-Elemene	204.35	3	0	0	4.53	1	Low	No	No
β-Levantenolide	318.45	0	3	0	4.16	1	High	No	No
