# Supplementary material for: HIV-1 Protease and Reverse Transcriptase Inhibitory Activities of Curcuma aeruginosa Roxb. Rhizome Extracts and the Phytochemical Profile Analysis: In Vitro and In Silico Screening
Source: Pharmaceuticals (Basel). 2021 Oct 31;14(11):1115. doi: 10.3390/ph14111115 (PMC8621417; doi:10.3390/ph14111115)
Supplement: Supplementary file 1 [file pharmaceuticals-14-01115-s001.zip › Supplementary data 5.pdf]

Supplementary data 5

Table S5 Molecular docking results of CA-identified compounds at the active site of HIV-1 PR

**Compound**	**Binding energy (kcal/mol)**	**Inhibition Constant**
Amprenavir	-9.73	73.84 nM
Dihydroergocornine	-12.65	537.84 pM
27-nor-5β-Cholestane-3α,7α,12α,24,25-pentol	-11.53	3.51 nM
3β,6α,7α-Trihydroxy-5β-cholan-24-oic acid	-10.92	9.82 nM
6β,11β,16α,17α,21-Pentahydroxypregna-1,4-diene-3,20-dione-16,17-acetonide	-10.71	14.15 nM
β-Levantenolide	-9.52	105.94 nM
Deoxysappanone B 7,3'-dimethyl ether acetate	-7.96	1.47 μM
Xanthumin	-7.84	1.8 μM
Punctaporin B	-7.67	2.38 μM
Dihydrocostunolide	-7.51	3.15 μM
Prostaglandin H1	-7.45	3.45 μM
Arglabin	-7.31	4.41 μM
QH2	-7.30	4.49 μM
Cycloisolongifolene,8,9-dehydro-9-formyl-	-7.23	5.01 μM
Lactone of PGF-MUM	-7.13	5.97 μM
6-(3-Hydroxyprop-1-en-2-yl)-4,8a-dimethyl-1,3,5,6,7,8-hexahydronaphthalen-2-one	-7.13	5.94 μM
Cadinol T	-7.10	6.28 μM
α-Cadinol	-7.09	6.39 μM
9-Isopropyl-1-methyl-2-methylene-5-oxatricyclo[5.4.0.03,8]undecane	-7.08	6.5 μM
Phe Ala Pro	-7.00	7.39 μM
Isoaromadendrene epoxide	-7.00	0.44 μM
4-(3,3-dimethylbut-1-ynyl)-4-hydroxy-2,6,6-trimethylcyclohex-2-en-1-one	-6.99	7.52 μM
Prostaglandin F1a alcohol	-6.96	7.9 μM
3-(3,3,8,8-Tetramethyl-5-tricyclo[5.1.0.02,5]oct-5-enyl)propanoic acid	-6.95	7.99 μM
Gemfibrozil M1	-6.93	8.28 μM
4,7,7-Trimethyl-4-(2-methylallyl)tricyclo[3.3.0.02,8]octane-3,6-dione	-6.84	9.61 μM
Gemfibrozil	-6.83	9.87 μM
(4Z)-4-(6,6-dimethyl-2-methylidenecyclohex-3-en-1-ylidene)pentan-2-ol	-6.71	12.15 μM
Gemfibrozil M3	-6.33	22.89 μM
Hydroxyibuprofen	-6.29	24.63 μM
Benzenehexanoic acid, 2,5-dihydroxy-3,4-dimethoxy-6-methyl-	-6.23	27.24 μM

Amiloxate	-6.13	31.84 µM
Ile Leu Leu	-6.09	34.39 µM
Phytosphingosine	-5.56	84.14 µM
Oleic Acid	-5.56	83.38 µM
Palmitic acid	-5.46	99.83 µM
Dihydrosphingosine	-5.38	114.19 µM
Methyl jasmonate	-5.37	116.31 µM
N-(2-hydroxyethyl) icosanamide	-5.35	118.85 µM
α-Terpineol	-5.31	128.56 µM
Dihydrojasmonic acid, methyl ester	-5.30	130.11 µM
3-n-Decyl acrylic acid	-5.28	134.41 µM
Citronellic acid	-5.08	118.65 µM
Val Val	-5.07	191.19 µM
Linoleic acid, methyl ester	-5.06	194.31 µM
Hexadecasphinganine	-4.98	222.36 µM
3-oxo-Tridecanoic acid	-4.95	233.85 µM
10-keto Tridecanoic acid	-4.94	240.31 µM
Pro Glu	-4.92	245.µM
Ile Thr	-4.87	270.30 µM
2-oxo-Dodecanoic acid	-4.86	275.66 µM
4-Hydroxy capric acid	-4.73	339.32 µM
7E,9Z-Dodecadien-1-ol	-4.69	363.46 µM
13-Hydroxy-tridecanoic acid	-4.64	406.37 µM
3-Dodecynoic acid	-4.41	585.68 µM
9-Dodecen-1-ol	-4.39	607.89 µM
4Z-Decenedioic acid	-4.23	799.57 µM
12-Hydroxy-10-octadecynoic acid	-4.19	850.84 µM
(E)-2-Methylglutaconic acid	-4.11	963.31 µM
Pantoic acid	-3.87	1.45 mM
Undecanal	-3.83	1.56 mM
2-Hydroxyethanesulfonate	-3.54	2.56 mM
4-Heptanone	-3.45	2.97 mM
Leucine	-3.39	3.28 mM
Octanal	-3.31	3.78 mM
Ethyl oxalacetate	-3.29	3.89 mM
4-Methylpentanal	-3.23	4.29 mM
Deoxyribose	-3.23	4.28 mM
3-Tridecynoic acid	-3.15	4.91 mM
Taurine	-2.96	6.79 mM
