# Supplementary material for: HIV-1 Protease and Reverse Transcriptase Inhibitory Activities of Curcuma aeruginosa Roxb. Rhizome Extracts and the Phytochemical Profile Analysis: In Vitro and In Silico Screening
Source: Pharmaceuticals (Basel). 2021 Oct 31;14(11):1115. doi: 10.3390/ph14111115 (PMC8621417; doi:10.3390/ph14111115)
Supplement: Supplementary file 1 [file pharmaceuticals-14-01115-s001.zip › Supplementary data 6.pdf]

Supplementary data 6

Table S6 Molecular docking results of CA-identified compounds at the DNA polymerase active site of HIV-1 RT

**Compound**	**Binding energy (kcal/mol)**	**Inhibition constant**
Nevirapine (original inhibitor)	-9.31	150.92 nM
3β,6α,7α-Trihydroxy-5β-cholan-24-oic acid	-10.39	24.31 nM
27-nor-5β-Cholestane-3α,7α,12α,24,25-pentol	-9.99	47.36 µM
Xanthumin	-9.73	73.3 nM
Prostaglandin F1a alcohol	-9.72	75.09 nM
QH2	-9.69	79.35 nM
Prostaglandin H1	-9.50	108.88 nM
Lactone of PGF-MUM	-9.48	112.42 nM
Dihydrocostunolide	-9.24	169.31 nM
Cadinol T	-9.22	173.56 nM
Arglabin	-9.22	174.13 nM
α-Cadinol	-9.20	181.51 nM
β-Levantenolide	-9.10	213.92 nM
6-(3-Hydroxyprop-1-en-2-yl)-4,8a-dimethyl-1,3,5,6,7,8-hexahydronaphthalen-2-one	-8.72	403.29 nM
Isoaromadendrene epoxide	-8.68	435.25 nM
Cycloisolongifolene,8,9-dehydro-9-formyl-	-8.64	467.89 nM
3-(3,3,8,8-Tetramethyl-5-tricyclo[5.1.0.02,5]oct-5-enyl)propanoic acid	-8.51	576.14 nM
(4Z)-4-(6,6-dimethyl-2-methylidenecyclohex-3-en-1-ylidene)pentan-2-ol	-8.36	750.58 µM
9-Isopropyl-1-methyl-2-methylene-5-oxatricyclo[5.4.0.03,8]undecane	-8.35	754.57 nM
Benzenehexanoic acid, 2,5-dihydroxy-3,4-dimethoxy-6-methyl-	-8.26	878.93 nM
4-(3,3-dimethylbut-1-ynyl)-4-hydroxy-2,6,6-trimethylcyclohex-2-en-1-one	-8.07	1.21 µM
Gemfibrozil	-8.04	1.28 µM
Gemfibrozil M1	-8.00	1.38 µM
Dihydroergocornine	-7.91	1.59 µM
Oleic Acid	-7.81	1.88 µM
4,7,7-Trimethyl-4-(2-methylallyl)tricyclo[3.3.0.02,8]octane-3,6-dione	-7.76	2.06 µM
Ile Leu Leu	-7.72	2.19 µM
6β,11β,16α,17α,21-Pentahydroxypregna-1,4-diene-3,20-dione-16,17-acetonide	-7.61	2.63 µM
Amiloxate	-7.53	3.04 µM
Punctaporin B	-7.49	3.21 µM

Linoleic acid, methyl ester	-7.45	3.43 µM
Methyl jasmonate	-7.40	3.74 µM
Dihydrosphingosine	-7.39	3.81 µM
Dihydrojasmonic acid, methyl ester	-7.15	5.71 µM
Palmitic acid	-7.13	5.91 µM
Hydroxyibuprofen	-7.13	5.99 µM
α-Terpineol	-6.66	13.21 µM
2-oxo-Dodecanoic acid	-6.62	14.09 µM
N-(2-hydroxyethyl) icosanamide	-6.54	15.95 µM
7E,9Z-Dodecadien-1-ol	-6.48	17.82 µM
Phytosphingosine	-6.47	18.12 µM
13-Hydroxy-tridecanoic acid	-6.46	18.46 µM
3-Dodecynoic acid	-6.38	21.07 µM
12-Hydroxy-10-octadecynoic acid	-6.19	29.14 µM
10-keto Tridecanoic acid	-6.16	30.78 µM
3-oxo-Tridecanoic acid	-6.13	32 µM
Hexadecasphinganine	-6.11	33.13 µM
4-Hydroxy capric acid	-5.95	43.86 µM
Gemfibrozil M3	-5.75	60.74 µM
Citronellic acid	-5.74	61.83 µM
Pro Glu	-5.52	90.04 µM
4Z-Decenedioic acid	-5.51	90.68 µM
Undecanal	-5.48	96.34 µM
Ile Thr	-5.46	99.34 µM
Val Val	-5.19	155.8 µM
3-n-Decyl acrylic acid	-5.08	188.25 µM
Ethyl oxalacetate	-4.75	329.23 µM
Octanal	-4.49	512.12 µM
(E)-2-Methylglutaconic acid	-4.30	703.07 µM
4-Heptanone	-4.27	741.7 µM
3-Tridecynoic acid	-4.16	887.83 µM
Pantoic acid	-4.04	1.09 mM
Deoxyribose	-4.00	1.17 mM
9-Dodecen-1-ol	-3.94	1.3 mM
4-Methylpentanal	-3.91	1.36 mM
Leucine	-3.65	2.21 mM
2-Hydroxyethanesulfonate	-3.29	3.87 mM
Taurine	-2.74	9.86 mM
