# Supplementary material for: HIV-1 Protease and Reverse Transcriptase Inhibitory Activities of Curcuma aeruginosa Roxb. Rhizome Extracts and the Phytochemical Profile Analysis: In Vitro and In Silico Screening
Source: Pharmaceuticals (Basel). 2021 Oct 31;14(11):1115. doi: 10.3390/ph14111115 (PMC8621417; doi:10.3390/ph14111115)
Supplement: Supplementary file 1 [file pharmaceuticals-14-01115-s001.zip › Supplementary data 7.pdf]

**Supplementary data 7**

**Table S7** Molecular docking results of CA-identified compounds at the RNase H active site of HIV-1 RT

| Compound | Binding energy (kcal/mol) | Inhibition constant |
|---|---|---|
| P4Y (original inhibitor) | -5.29 | 132.5 µM |
| 3β,6α,7α-Trihydroxy-5β-cholan-24-oic acid | -6.77 | 10.83 µM |
| 27-nor-5β-Cholestane-3α,7α,12α,24,25-pentol | -6.58 | 15.11 µM |
| Hydroxyibuprofen | -5.92 | 45.54 µM |
| 6β,11β,16α,17α,21-Pentahydroxypregna-1,4-diene-3,20-dione-16,17-acetonide | -5.90 | 47.56 µM |
| Dihydroergocornine | -5.80 | 56.02 µM |
| Lactone of PGF-MUM | -5.57 | 82.02 µM |
| Pro Glu | -5.44 | 103.08 µM |
| β-Levantenolide | -5.38 | 114.1 µM |
| Val Val | -5.34 | 121.36 µM |
| 6-(3-Hydroxyprop-1-en-2-yl)-4,8a-dimethyl-1,3,5,6,7,8-hexahydronaphthalen-2-one | -5.25 | 141.74 µM |
| 3-(3,3,8,8-Tetramethyl-5-tricyclo[5.1.0.02,5]oct-5-enyl)propanoic acid | -5.17 | 161.15 µM |
| Gemfibrozil M1 | -5.16 | 165.59 µM |
| Gemfibrozil | -5.13 | 174.67 µM |
| Xanthumin | -5.07 | 193.22 µM |
| Prostaglandin H1 | -5.05 | 198.21 µM |
| Cadinol T | -4.89 | 262.45 µM |
| Punctaporin B | -4.86 | 271.97 µM |
| Ile Thr | -4.80 | 301.79 µM |
| Dihydrocostunolide | -4.74 | 336.3 µM |
| α-Cadinol | -4.55 | 465.37 µM |
| Arglabin | -4.52 | 485.39 µM |
| 4Z-Decenedioic acid | -4.51 | 492.23 µM |
| 9-Isopropyl-1-methyl-2-methylene-5-oxatricyclo[5.4.0.03,8]undecane | -4.48 | 524.49 µM |
| (4Z)-4-(6,6-dimethyl-2-methylidenecyclohex-3-en-1-ylidene)pentan-2-ol | -4.45 | 543.19 µM |
| Gemfibrozil M3 | -4.45 | 545.78 µM |
| 2-Hydroxyethanesulfonate | -4.40 | 593.72 µM |
| (E)-2-Methylglutaconic acid | -4.38 | 612.4 µM |
| 4-(3,3-dimethylbut-1-ynyl)-4-hydroxy-2,6,6-trimethylcyclohex-2-en-1-one | -4.33 | 667.9 µM |
| Cycloisolongifolene,8,9-dehydro-9-formyl- | -4.30 | 709.1 µM |
| Benzenehexanoic acid, 2,5-dihydroxy-3,4-dimethoxy-6-methyl- | -4.30 | 704.81 µM |

| | | |
|---|---|---|
| Isoaromadendrene epoxide | -4.25 | 763.78 µM |
| Amiloxate | -4.24 | 782.22 µM |
| Citronellic acid | -4.22 | 807.0 µM |
| Phytosphingosine | -4.21 | 819.94 µM |
| 4,7,7-Trimethyl-4-(2-methylallyl)tricyclo[3.3.0.02,8]octane-3,6-dione | -4.14 | 931.07 µM |
| Ile Leu Leu | -4.14 | 917.91 µM |
| 13-Hydroxy-tridecanoic acid | -4.12 | 961.57 µM |
| 4-Hydroxy capric acid | -4.09 | 1.01 mM |
| Pantoic acid | -4.08 | 1.01 mM |
| QH2 | -4.08 | 1.02 mM |
| Hexadecasphinganine | -4.00 | 1.17 mM |
| α-Terpineol | -3.93 | 1.32 mM |
| 3-oxo-Tridecanoic acid | -3.71 | 1.92 mM |
| 3-n-Decyl acrylic acid | -3.66 | 2.06 mM |
| Dihydrosphingosine | -3.61 | 1.98 mM |
| 10-keto Tridecanoic acid | -3.58 | 2.37 mM |
| Prostaglandin F1a alcohol | -3.51 | 2.65 mM |
| Methyl jasmonate | -3.49 | 2.78 mM |
| Taurine | -3.43 | 3.06 mM |
| 3-Dodecynoic acid | -3.38 | 3.34 mM |
| Palmitic acid | -3.37 | 3.36 mM |
| Leucine | -3.29 | 3.85 mM |
| Oleic Acid | -3.23 | 4.32 mM |
| 2-oxo-Dodecanoic acid | -3.07 | 5.62 mM |
| Deoxyribose | -3.06 | 5.72 mM |
| Dihydrojasmonic acid, methyl ester | -3.04 | 5.93 mM |
| 7E,9Z-Dodecadien-1-ol | -2.78 | 9.14 mM |
| Ethyl oxalacetate | -2.74 | 9.8 mM |
| 9-Dodecen-1-ol | -2.53 | 14.0 mM |
| 4-Heptanone | -2.52 | 14.22 mM |
| 4-Methylpentanal | -2.51 | 14.37 mM |
| Octanal | -2.33 | 19.45 mM |
| Undecanal | -2.18 | 25.1 mM |
| Linoleic acid, methyl ester | -1.65 | 61.56 mM |
| 12-Hydroxy-10-octadecynoic acid | -1.43 | 89.21 mM |
| 3-Tridecynoic acid | -0.91 | 213.6 mM |
| N-(2-hydroxyethyl) icosanamide | -0.49 | 439.99 mM |
